# *Massospondylus* embryos and hatchling provide new insights into early sauropodomorph ontogeny

**DOI:** 10.1186/s13358-025-00382-5

**Published:** 2025-08-04

**Authors:** Ethan D. Mooney, Tea Maho, Dylan C. T. Rowe, Diane Scott, Robert R. Reisz

**Affiliations:** 1 Department of Biology, University of Toronto Mississauga, 3359 Mississauga Rd., Mississauga, ON L5l 1C6 Canada; 2 Dinosaur Evolution Research Center, International Center of Future Science, Jilin University, 2699 Qianjin Str., Changchun, 130012 Jilin China

## Abstract

Ontogeny and patterns of growth provide crucial insights into the evolution of dinosaurs and their biology, however, ontogenetic changes of the postcranial skeleton are generally poorly known and restricted to few species. Here, we report on the discovery of embryonic, eggshell, and hatchling material of the Early Jurassic sauropodomorph *Massospondylus* and provide new insights into the early life stages of early-diverging sauropodomorphs. The embryos described here represent a more advanced developmental stage than previously known embryonic materials and together show eggshells with progressive degrees of resorption correlating to embryonic development. The prominent “thumb” typical of early-diverging sauropodomorph dinosaurs also appears to be precociously ossified and ontogenetically variable. Together, these new *Massospondylus* embryos and hatchling indicate a quadrupedal posture and the resulting growth trajectory points to an important ontogenetically influenced ecological shift predicated on a continuum of locomotory dependent postural change, from obligate quadrupeds to obligate bipeds*. *Other Late Triassic and Early Jurassic sauropodomorph dinosaurs known from few ontogenetic stages also fit well on the extensive growth trajectory of *Massospondylus* with only modest variations in skull, neck, and limb proportions. This generally conservative body plan of early-diverging sauropodomorphs likely suggests a similar postural change for the early members of this large clade that preceded the obligate quadruped sauropods.

**Supplementary Information:**

The online version contains supplementary material available at 10.1186/s13358-025-00382-5.

## Introduction

Ontogenetic variation in dinosaurs and other extinct vertebrates remains a topic of great significance and debate in paleontology. Fossils representing the early life stages of dinosaurs reveal vital insights into their complex development, ecologies, reproduction, and evolution. However, the fossil record is strongly skewed towards the preservation of late-ontogenetic-stage juveniles, subadults, and adults, while embryos and hatchlings are rare and largely known from the Cretaceous Period (Carpenter & Alf, 1994; Long & McNamara, 1995; Chiappe et al., 1998; Kundrát et al., 2008; Stanford et al., 2011; Zhao et al., 2013; Lee et al., 2019; Funston et al., 2021). As a result, a substantial lack of understanding surrounds the embryonic and hatchling stages of many early dinosaurian groups, but in recent years, discoveries have made some strides in filling this knowledge gap, specifically those of early-diverging sauropodomorphs.

Early-diverging sauropodomorph dinosaurs include some of the earliest-known dinosaurs and they made up significant components of most terrestrial ecosystems in the Early Jurassic (Brusatte et al., 2008, 2010; Galton & Upchurch, 2004; McPhee et al., 2017). They were already the most numerous, diverse, and globally distributed dinosaurs by the end of the Triassic Period, and their success has been attributed to their large body sizes, a shift to herbivory, rapid growth rates, herding, and various unique anatomies (Apaldetti et al., 2018; Barrett, 2014; Barrett & Upchurch, 2005, 2007; Benson et al., 2014; Galton & Upchurch, 2004; Pol et al., 2021). However, despite the preservation of numerous Late Triassic and Early Jurassic sauropodomorphs, their early ontogenetic stages are seldom preserved, which makes the extensive ontogenetic series of the Early Jurassic genus *Massospondylus* from Southern Africa particularly significant (Chinsamy, 1993; Reisz et al., 2005, 2010, 2012; Neenan et al., 2019; Chapelle et al., 2020a, 2020b, 2021, 2022).

*Massospondylus carinatus* Owen, 1854 is an iconic and well-studied sauropodomorph dinosaur (e.g., Cooper, 1981; Kitching & Raath, 1984; Galton & Upchurch, 2004; Sues et al., 2004; Barret et al., 2019) from the Elliot and Clarens formations (*Massospondylus* Range Zone) (Viglietti et al., 2020), but also the Forest Sandstone Formation from the Mid-Zambezi Basin of Southern Africa (Sciscio et al., 2021). Fossilized embryos recovered from eggs, as well as juvenile and adult individuals, have revealed substantial ontogenetic and evolutionary discoveries for *Massospondylus* and other early-diverging sauropodomorphs (e.g., Cooper, 1981; Kitching et al., 1984; Gow, 1990; Chinsamy, 1993; Galton & Upchurch, 2004; Sues et al., 2004; Reisz et al., 2005, 2010, 2012, 2013, 2020, 2024; Bonnan & Senter, 2007; Yates et al., 2010; Barret et al., 2019; Neenan et al., 2019; Otero et al., 2019; Chapelle et al., 2020a, 2020b, 2021, 2022; Nau et al., 2020; Otero & Pol, 2021; Pol et al., 2021; Cerda et al., 2022; Han et al., 2024). Here, we add to this understanding by reporting on embryos and a hatchling individual attributable to *Massospondylus *sp. that provide critical new developmental information and new insights into the ontogeny, evolution, and locomotory-influenced ecology of sauropodomorph dinosaurs.

## Materials and methods

### Specimen information

Mechanical preparation of all new *Massospondylus* material was performed under a Leica MZ6 dissecting microscope using various miniature pneumatic tools including a pin vice with carbide rods.

Two previously undescribed *Massospondylus* embryos BP/1/5346 are within eggs and were collected from the base of the famous Rooidraai roadside exposure of the upper Elliot Formation within the Golden Gate Highlands National Park, South Africa, during previous excavations but remained unprepared and unstudied. They were collected by the late James W. Kitching along the base of the same roadcut as the *Massospondylus* clutch BP/1/5347 and originate at the level identified as clutch N8 by Reisz et al. (2012). Since the materials came from a near vertical exposure and were located slightly higher in the exposure than the more accessible clutches, only two eggs were collected while the rest of the clutch remains in situ. They are embedded within the same reddish-brown, muddy siltstone matrix as the *Massospondylus* clutch BP/1/5347 (Reisz et al., 2005, 2010) and the nest described by Reisz et al. (2012). Sediments of this locality are considered indicative of an ephemeral lake shore or edge of a large meandering river channel, likely an oxbow lake prone to flood burial events (Reisz et al., 2012).

The hatchling SAM-PK-K413 is tentatively assigned to *Massospondylus *sp. based on the anatomical features discussed here, most notable of which is the enlarged thumb claw and that of the cervical vertebrae (see Anatomical Description of *Massospondylus* Hatchling). This hatchling is recorded as having been recovered from the Early Jurassic Stormberg group of the Elliot formation, where the most common dinosaur is also *Massospondylus carinatus*. It was an isolated specimen collected by the British-South African Palaeontological Expedition in 1961 from site 48 of the Likhoele farm, Mafeteng, Lesotho, although there is some doubt as to the accuracy of this locality data. This specimen was also on display at the Iziko Museum until 2003, initially having been misidentified as *Heleosaurus*.

### Growth trajectory

A growth trajectory for *Massospondylus *sp. has been previously constructed (Reisz et al., 2005), for which we have now added two critically important early ontogenetic stages (the first embryo of BP/1/5346 and hatchling SAM-PK-K413). We have also added three additional types of measurements to this data set (maximum scapular length, minimum scapula shaft width, and maximum scapular blade width) because of their relevance in evaluating posture and locomotory ability.

Measurements of other early-diverging sauropodomorphs were obtained directly from personal observations of the senior author, the literature, or photographs therein. All analyses were performed with RStudio 2021.09.2 (RStudio Team, 2021) and the graphs were constructed using the ‘ggplot2’ R package (Wickham, 2016).

### Institutional abbreviations

BP: Evolutionary Studies Institute (ESI: formerly Bernard Price Institute), University of the Witwatersrand, Johannesburg, South Africa

CAPPA/UFSM: Centrode Apoio à Pesquisa Paleontológica da Quarta Colônia da Universidade Federal de Santa Maria, São João do Polêsine, Brazil

GSI/GC: Geological Survey of India Palaeontology Division, Central Region, Nagpur, India

GZPM: Guizhou Provincial Museum, Guiyang, People’s Republic of China

ISI: Indian Statistical Institute, Kolkata, India

IVPP: Institute of Vertebrate Paleontology and Paleoanthropology, Beijing, People’s Republic of China

MACN: Museo Argentino de Ciencias Naturales ‘Bernardino Rivadavia’, Buenos Aires, Argentina

MB: Institut für Paläontologie, Museum fur Naturkunde, Humbolt-Universität, Berlin, Germany

MCP: Museu de Ciências e Tecnologia Pontifícia Universidade Católica do Rio Grande do Sul, Porto Alegre, Brazil

MSF: Sauriermuseum Frick, Frick, Switzerland

PVL: Instituto ‘Miguel Lillo’, Tucumán, Argentina

PVSJ: Museo de Ciencias Naturales, Universidad Nacional de San Juan, San Juan, Argentina

SAM-PK: South African Museum, Cape Town, South Africa

SMNS: South African Museum, Cape Town, South Africa

TMM: Texas Memorial Museum, Austin, Texas, U.S.A.

YPM: Yale Peabody Museum, Yale University, New Haven, Connecticut, U.S.A.

## Results

### Anatomical description of *Massospondylus* embryos

**Fig. 1:**
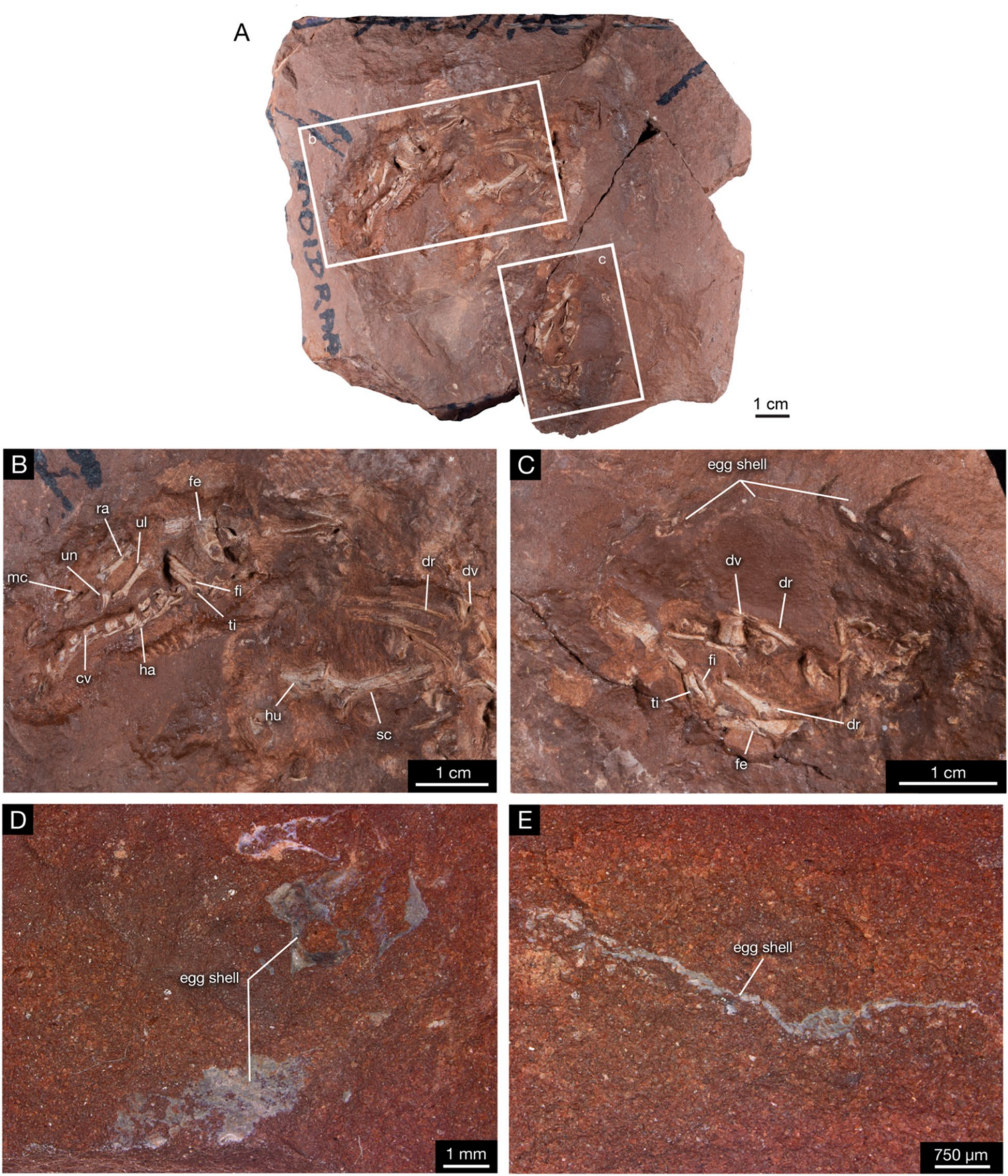
*Massospondylus *sp. embryos BP/1/5346. **A** Photograph of the complete block. **B** Close-up photograph of the postcranial elements of the first embryo. **C** Close-up photograph of the cranial and postcranial material and eggshell of the second embryo. **D** Photograph of eggshell and **E** Photograph of eggshell cross section. *cv* caudal vertebra, *dr* dorsal rib, *dv* dorsal vertebra, *fe* femur, *fi* fibula, *ha* hemal arch, *hu* humerus, *mc* metacarpal, *ra* radius, *sc* scapula, *ti* tibia, *ul* ulna, *un* ungual.

The block BP/1/5346 contains a pair of embryos still within the egg (Fig. 1A), one largely represented by postcranial material (Fig. 1B) and the other predominantly by cranial material (Fig. 1C). The embryos are attributable to *Massospondylus *sp. like that of other embryonic remains from this site. Despite the absence of identifiable cranial elements, these skeletons are anatomically indistinguishable from the previously described embryos from this taxon (BP/1/5347; Reisz et al., 2005, 2010).

The first embryonic skeleton of BP/1/5346 appears articulated in a fetal posture reminiscent of BP/1/5347A, in which the tail and forelimbs are tucked between the hind limbs and are thereby largely obscured from view (Fig. 1B). It consists of an articulated caudal series with hemal arches, an exposed radius, ulna, and manus with a well-ossified thumb claw, all of which are consistent with the known embryonic anatomies of *Massospondylus* BP/1/5347. The manus is positioned distal to the radius and ulna, and includes a prominent thumb claw, phalanges, and metacarpals. Additionally, there is a partially articulated thoracic region with thoracic ribs exposed in association with dorsal vertebrae, a floating humerus, and a scapula as well. The second embryonic skeleton of BP/1/5346 is largely disarticulated cranial material that is extremely fragile and fragmented, as well as associated dorsal vertebra consistent with BP/1/5347A (Fig. 1C). The outer boundary of the egg is denoted by the thin eggshell identical in structure and surface texture to previously described *Massospondylus* eggshell from the same locality (Stein et al., 2019). The eggshell is extremely thin with variation in thickness between 38 and 56 µm, which was measurable directly from the exposed eggshell cross section (Fig. 1D, E).

### Anatomical description of *Massospondylus* hatchling

**Fig. 2:**
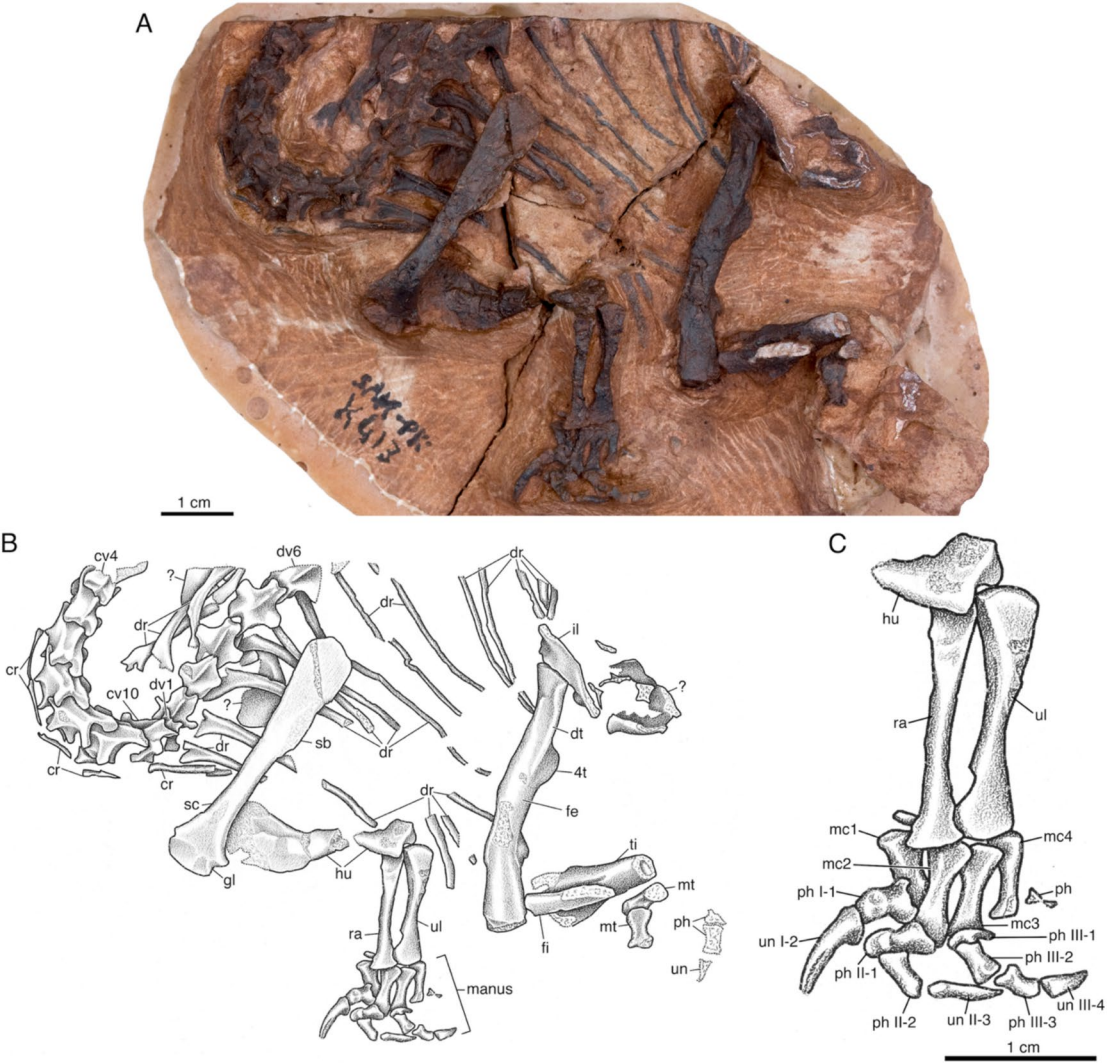
*Massospondylus *sp. hatchling SAM-PK-K413. **A** Complete specimen photograph, **B** complete specimen illustration, and **C** close-up of left forelimb illustration. *cv* cervical vertebra, *cr* cervical rib, *dr* dorsal rib, *dt* dorsolateral trochanter, *dv* dorsal vertebra, *fe* femur, *fi* fibula, *gl* glenoid, *hu* humerus, *il* ilium, *mc* metacarpal, *mt* metatarsal, *ph* phalange, *ra* radius, *sc* scapula, *ti* tibia, *ul* ulna, *un* ungual, *4t* 4th trochanter

SAM-PK-K413 is a largely articulated postcranial skeleton consisting of several cervical and dorsal vertebrae with their associated ribs, a nearly complete left forelimb along with pelvic fragments, and a partial left hindlimb (Fig. 2). This individual is clearly a hatchling based on its small size, undeveloped articulating surfaces of the limb bones, and the lack of ossified elements like the carpals and tarsals. The entire skeleton is also encrusted with what appears to be a dark brown-black ferrous or manganese oxide, which is typical of fossils from the Upper Elliot Formation.

There are several features of SAM-PK-K413 that are consistent with *Massospondylus*, most notable are those of the cervical vertebrae and the well-developed thumb claw. Nearly the complete cervical series is represented. The fourth through tenth cervical vertebrae are visible in dorsal view. The centra of the cervical vertebrae are spool-shaped and slightly elongated centra compared to the dorsal vertebrae, as is noted in *Massospondylus* embryonic material BP/1/5347 (Reisz et al., 2005, 2010). Each neural spine successively increases in height and projects posterodorsally. This condition is most prominent at the eighth cervical vertebrae before shortening dramatically in the ninth and tenth cervical vertebrae, which is also observed in adult *Massospondylus* skeletons (Fig. S1) (Barret et al., 2019). The zygapophyses also exhibit the following similar morphological variation throughout the cervical series consistent with that of *Massospondylus* (Fig. S1) (Barret et al., 2019; Reisz et al., 2005, 2010). The prezygapophyses gradually decrease in anteroposterior length throughout the cervical series. The postzygapophyses of the fourth and fifth cervical vertebrae extend posterolaterally and are noticeably short and stout with a rounded terminus. Those of the sixth through eighth cervical vertebrae become successively more elongated, with the mediolateral length of those belonging to the eighth cervical being equal to that of the transverse processes. As expected for the posterior-most cervicals and as in well-preserved adult *Massospondylus* specimens, the postzygapophyses are markedly shortened among vertebrae transitioning into the thoracic region (Figs. S1, S2, S3, S4) (Barret et al., 2019). The transverse processes of the cervical vertebrae also successively increase in length and width, noticeably starting at the sixth cervical vertebra to accommodate ever more robust cervical ribs. Slender cervical ribs are associated with the fifth through eighth cervical vertebrae, while cervical ribs associated with the ninth and tenth cervical vertebrae become more robust to more closely resemble the dorsal ribs.

There are six dorsal vertebrae preserved, each with their respective dorsal ribs. The neural spines of the dorsal vertebrae are substantially truncated relative to those of the cervical vertebrae and remain uniform throughout the series without extending past the postzygapophyses. The transverse processes successively broaden anteroposteriorly and extend laterally to support the dorsal ribs. The prezygapophyses and postzygapophyses retain a typical morphology consistent throughout the dorsal series of adult *Massospondylus* (Figs. S2, S3, S4) (Barret et al., 2019). While most dorsal ribs are incomplete, a well-preserved tuberculum and capitulum are present for each. It is not possible to determine the level of co-ossification between the neural arches and centra. The partial exposure of the centrum of the third dorsal vertebra in the hatchling SAM-PK-K413 also shows greater co-ossification than those of the embryonic materials BP/1/5346 and BP/1/5347.

The left arm is partially articulated in a pronated position and consists of the left scapula with no identifiable coracoid, humerus, ulna, radius, and manus lacks carpals (Fig. 2C). Only the left scapula is exposed, with no identifiable coracoid. The scapula is slender like the embryonic condition of *Massospondylus* embryos BP/1/5347 (Reisz et al., 2005, 2010), as opposed to the broad condition exemplified by mature individuals, including BP/1/4934 (Fig. S2) (Barret et al., 2019). However, it differs from the embryonic condition shown by BP/1/5347 in that the distal end of the scapular blade is roughly twice the width of the midshaft, which reflects its slightly more mature ontogenetic stage.

A slightly damaged left humerus is articulated with the scapula, ulna and radius. The proximal end of the humerus is wider than the distal end due to the deltopectoral crest, which is consistent with the embryonic material BP/1/5347 (Reisz et al., 2005, 2010). While part of the midshaft and a portion of the deltopectoral crest are missing, the proximal and distal ends remain in-situ and it measures 27.1 mm in total length.

The anatomy and level of ossification of the radius and ulna are also consistent with the condition of previously reported embryonic material BP/1/5347A (Reisz et al., 2005, 2010). The manus is partially articulated and nearly all bones are preserved except the fifth digit. As with the previously described embryo BP/1/5347A, the carpals have not been preserved, likely due to their incomplete ossification typical of the juvenile condition (Reisz et al., 2005, 2010). Some postmortem disarticulation has shifted the metacarpals towards the distal end of the radius and ulna, with the first metacarpal lying underneath the second. The first metacarpal is the most robust, followed by the similarly sized second and third metacarpals, and then the fourth metacarpal, which is the most slender. The first digit possesses one proximal phalanx and an elongated, large ungual, while the second and third digits have two proximal phalanges each. Additionally, a delicate proximal phalanx is present, likely belonging to the fourth digit. Consistent with the embryonic and adult conditions (Fig. S3) of *Massospondylus*, the first ungual is equal in length to that of the largest metacarpal, although its distal tip is missing. The first ungual is dramatically larger than all other corresponding unguals and measures 6.6 mm long, which is nearly 1.5× larger than the second ungual measuring 4.7 mm, and is nearly 2.4× larger than the third ungual measuring 2.8 mm long (Fig. 2C). Furthermore, the first ungual is modestly curved, elongated, and slender, with a width of 1 mm, while the other two unguals appear to be nearly straight.

The left hindlimb is partially preserved and consists of a femur and the proximal portions of the tibia and fibula. Several fragments of the pelvic girdle are also visible and what is likely part of the ilium appears to articulate with the proximal end of the femur. The femur is approximately 38 mm long and 4 mm wide at the midshaft. A dorsolateral trochanter is visible and extends from the proximal head of the femur to the level of the fourth trochanter. An additional flange is also present towards the distal head and likely represents the beginning of the distal expansion of the femur. Only the proximal head of the tibia and a small proximal portion of the fibula remain. The likely remains of two pedal digits are also represented by two metatarsals and a weathered set of two articulated phalanges with an ungual.

### *Massospondylus* ontogeny

**Fig. 3:**
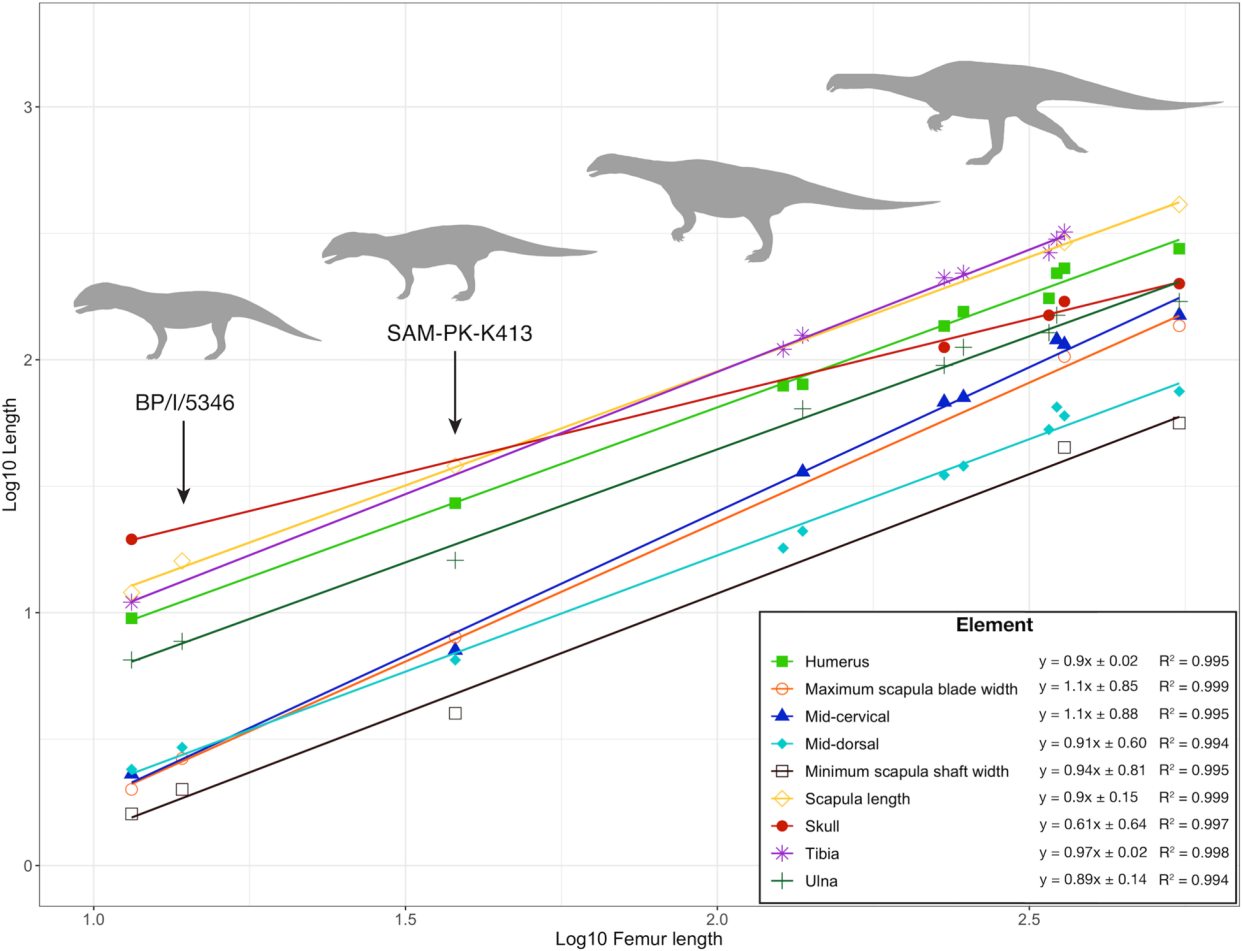
Expanded growth trajectory for *Massospondylus *sp. from Reisz et al. (2005) with new specimens (the first embryo of BP/1/5346 and hatchling SAM-PK-K413 are indicated by the *black arrows*) and addition of maximum scapular length, minimum scapula shaft width, and maximum scapular blade width measurements. This graph correlates Log10 femur length—Log10 length of various skeletal elements for eleven *Massospondylus* specimens. Allometric coefficients from regression analyses are provided for each skeletal element in the legend. See Supplementary Data 1, Table 1 for element measurements.

These new embryonic (BP/1/5346) and hatchling (SAM-PK-K314) specimens add to our limited understanding of the earliest ontogenetic stages of *Massospondylus* (Fig. 3) (Reisz et al., 2005, 2010; Neenan et al., 2019; Chapelle et al., 2020b). The skull, ulna, and humerus have negative allometric coefficients indicating a reduced growth rate of these skeletal elements compared to the femur. However, the cervical vertebrae and distal scapular blade width have positive allometries, with the strongest being that of the cervical vertebrae. Lastly, the dorsal vertebra, tibia, scapular length, and scapular shaft width have allometric coefficients closest to 1, indicating that these skeletal elements grew near isometrically with the femur.

### Ontogenetic comparisons among early-diverging sauropodomorphs

**Fig. 4:**
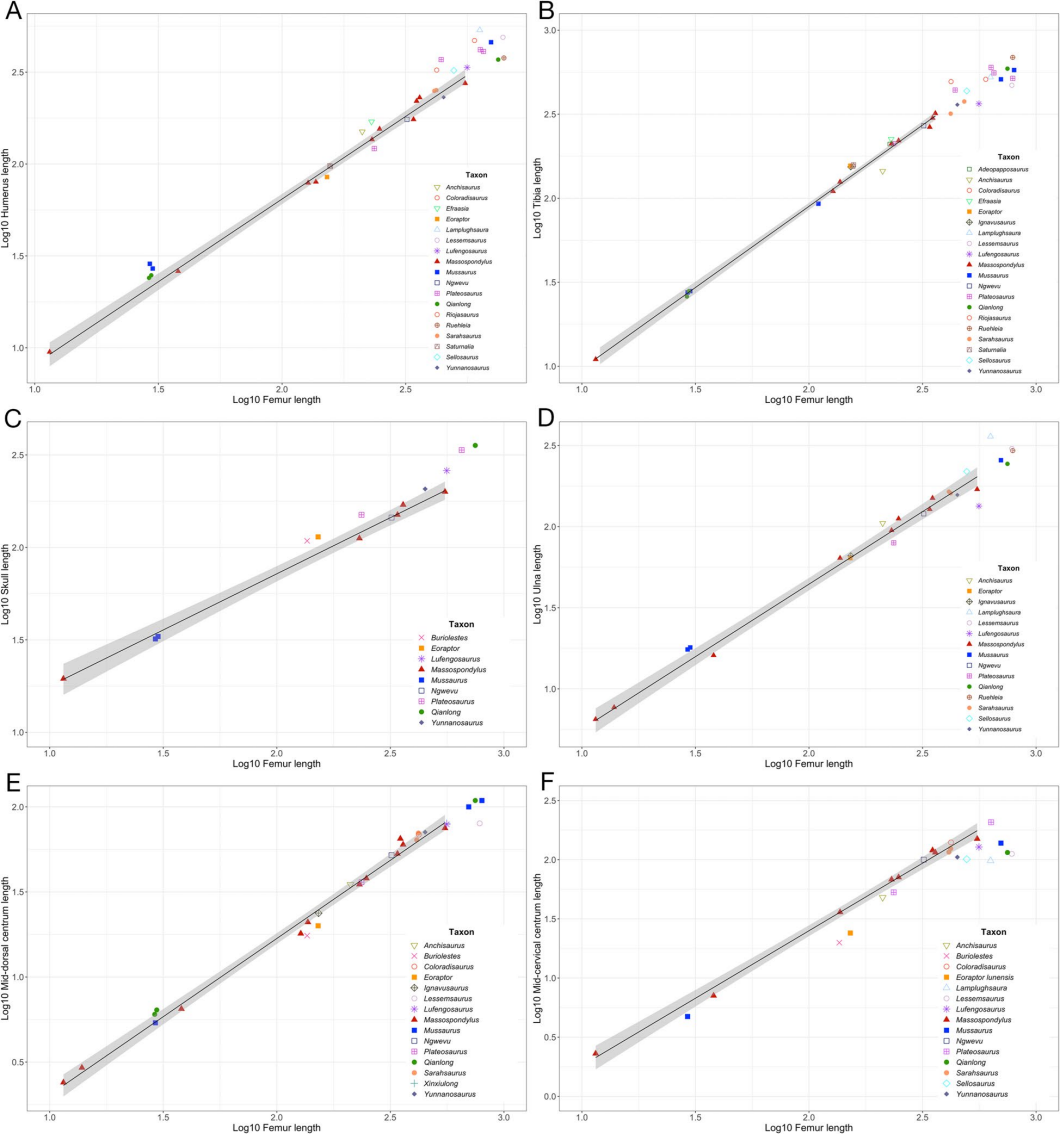
Early diverging sauropodomorph skeletal proportions plotted against Log femur lengths in relation to 95% confidence interval of *Massospondylus *sp. growth trajectory denoted in *grey*. **A** Humerus length; **B** tibia length; **C** skull length; **D** ulna length; **E** mid-dorsal centrum length; **F** mid-cervical centrum length. See Supplementary Data 1, Table 2 for element measurements and their respective references.

This comprehensive ontogenetic series of *Massospondylus* allows us to postulate patterns of growth not known in other Late Triassic and Early Jurassic sauropodomorphs. We compare the ontogenetic series of *Massospondylus* with twenty-two other sauropodomorph dinosaurs to investigate ontogenetically influenced body proportions for taxa with little or no ontogenetic information (Fig. 4). Thus, we can test where other morphologically similar sauropodomorphs plot along the growth trajectory of *Massospondylus*. We recognize the limitations of this approach since the ontogenetic stage represented by other sauropodomorphs may often not correspond to the same stage shown in *Massospondylus,* but the results provide important insights into the similarities and differences that characterize sauropodomorph dinosaurs as they increased in size and acquired sauropod body proportions.

The skull of hatchlings belonging to *Mussaurus* (Otero et al., 2019: PVL 4068; Otero & Pol, 2021: MACN-PV 4111) and that of the mature *Ngwevu intloko* (Chapelle et al., 2019: BP/1/4779) plot within the 95% confidence interval of the *Massospondylus* growth trajectory, whereas all other early-diverging sauropodomorph taxa included here, plot slightly above with relatively larger skull lengths than *Massospondylus* (Fig. 4C). *Buriolestes schultzi* (Müller et al., 2018: CAPPA/UFSM 0035) and *Eoraptor lunensis* (Sereno et al., 2013: PVSJ 512), the most basal presumed sauropodomorph taxa included, plot similarly above. It is notable that *Ngwevu intloko* (Chapelle et al., 2019: BP/1/4779), formerly considered to be part of the *Massospondylus* growth trajectory (Reisz et al., 2005), is the sole taxon to fit perfectly along the trend line of the 95% confidence interval of the *Massospondylus* growth trajectory.

The cervical vertebrae of *Ngwevu intloko* (Chapelle et al., 2019: BP/1/4779), *Coloradisaurus brevis* (Apaldetti et al., 2012: PVL5904), and *Sarahsaurus aurifontanalis* (personal observation: TMM 43646-56; Marsh & Rowe, 2018: TMM 43646-2) all plot within the 95% confidence interval of the *Massospondylus* growth trajectory (Fig. 4F). Interestingly, the Late Triassic *Plateosaurus gracilis* (Huene, 1907/1908: 53537)*,* which has a larger femur than any known specimen of *Massospondylus*, plots along this *Massospondylus* growth trajectory. All other taxa included in this analysis plot below the *Massospondylus* growth trajectory, including *Buriolestes schultzi* (Müller et al., 2018: CAPPA/UFSM 0035), *Eoraptor lunensis* (Sereno et al., 2013: PVSJ 512)*,* and *Lessemsaurus sauropoides* (Pol & Powell, 2007: PVL 4822). Neck-cervical elongation is a sauropodomorph condition, but most Early Jurassic taxa plot slightly below *Massospondylus*, indicating that *Massospondylus* had longer than average cervical vertebrae compared to many other well-known Early Jurassic sauropodomorphs tested here.

The dorsal vertebrae of most taxa plot either within the 95% confidence interval of the *Massospondylus* growth trajectory as expected, apart from *Buriolestes schultzi* (Müller et al., 2018: CAPPA/UFSM 0035), *Eoraptor lunensis* (Sereno et al., 2013: PVSJ 512), and *Lessemsaurus sauropoides* (Pol & Powell, 2007: PVL 4822) that plot slightly below. Overall, the ontogenetic relationship between femur length and mid-dorsal vertebral length appears to be nearly isometric, suggesting a close relationship between body mass and femur length (Fig. 4E). Interestingly, apart from *Coloradisaurus brevis* (Apaldetti et al., 2012: PVL 5904), Late Triassic taxa plot below that of *Massospondylus*.

The ulna is the most readily measurable element of the zeugopodium, but the ontogenetic level of ossification of the olecranon may be a confounding factor. *Mussaurus* hatchlings (Otero et al., 2019: PVL 4068; Otero & Pol, 2021: MACN-PV 4111) plot slightly above the *Massospondylus* confidence interval (Fig. 4D), but the adult *Mussaurus* (Otero & Pol, 2013: MLP 68-II-27-1A) and *Qianlong* (Han et al., 2024: GZPM VN001) individuals appears to have relatively longer ulna that are more consistent with that seen in *Massospondylus*. *Lufengosaurus huenei* (Young, 1941: IVPP V15) and material suggested to belong to a *Plateosaurus trossingensis* juvenile (Nau et al., 2020: MSF 15.8B) plot just below the 95% confidence interval of the *Massospondylus* growth trajectory indicating a relatively shorter ulna at adult size, while *Lamplughsaura dharmaramensis* (Kutty et al., 2007: ISI R257) plots far above. It is also worth noting that *Lessemsaurus sauropoides* (Pol & Powell, 2007: PVL 4822) and *Ruehleia bedheimensis* (Galton, 2001: MB RvL 1) plot very closely to each other and likely within the *Massospondylus* confidence interval if it were extended beyond the largest known individual.

The humerus for most taxa plot above the 95% confidence interval of the *Massospondylus* growth trajectory, especially among the larger known specimens, similar to or larger than the largest known specimen of *Massospondylus* (Fig. 4A)*.* The *Mussaurus* (Otero et al., 2019: PVL 4068; Otero & Pol, 2021: MACN-PV 4111) and *Qianlong* embryos (Han et al., 2024: GZPM VN006-1, GZPM VN006-2) both plot slightly above, with *Qianlong* slightly below that of *Mussaurus*, which are results consistent with that of the ulna. *Eoraptor lunensis* (Sereno et al., 2013: PVSJ 512) and the *Plateosaurus trossingensis* specimen suggested to be a juvenile (Nau et al., 2020: MSF 15.8B) plot slightly below the 95% confidence interval of the *Massospondylus* growth trajectory. However, within this distribution, there also appears to be a greater degree of variation in humeral length among differing, larger and more mature early-diverging sauropodomorphs. The distribution of many Early Jurassic taxa above the 95% confidence interval of the *Massospondylus* growth trajectory may also indicate the same trend, in that early-diverging sauropodomorphs of greater size and maturity have a comparatively greater humeral to femoral ratio than that of *Massospondylus*.

**Fig. 5:**
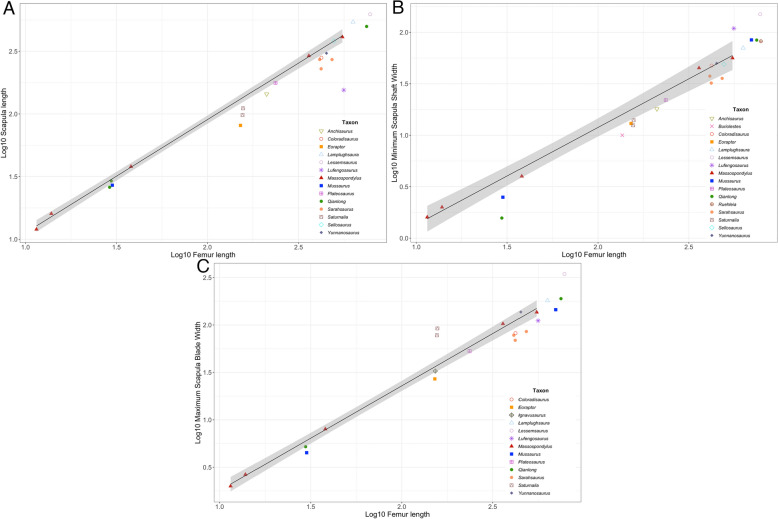
Early diverging sauropodomorph scapular proportions plotted against Log femur lengths in relation to 95% confidence interval of *Massospondylus *sp. growth trajectory denoted in *grey*. **A** Scapular length; **B** maximum scapular mid-shaft width; **C** Minimum scapular blade width. See Supplementary Data 1, Table 2 for element measurements and their respective references.

There is apparent ontogenetic and taxonomic variation in scapular morphologies in early-diverging sauropodomorphs from the Late Triassic and Early Jurassic (Fig. 5). The results suggest that for scapular length, most taxa plot below the 95% confidence interval of the *Massospondylus* growth trajectory, except for *Lamplughsaura dharmaramensis* (Kutty et al., 2007: ISI R257) and *Lessemsaurus sauropoides* (Pol & Powell, 2007: PVL 4822), which plot beyond and likely above (Fig. 5A). Interestingly, *Qianlong* (Han et al., 2024: GZPM VN001, GZPM VN004-2, GZPM VN006-1) and *Mussaurus* (Otero et al., 2019: PVL 4068) plot within or very close to the 95% confidence interval of the *Massospondylus* growth trajectory. *Sellosaurus gracilis* (Galton, 1984: SMNS 17928) also plots especially well on the *Massospondylus* growth trajectory. The minimum mid-shaft width of the scapula is more variable within *Massospondylus* and still many of the other early-diverging sauropodomorph taxa tested here plot outside the 95% confidence interval (Fig. 5B). For example, *Yunnanosaurus huangi* (Young, 1942: IVPP V20), *Coloradisaurus brevis* (Apaldetti et al., 2012: PVL 5904), *Sellosaurus gracilis* (Galton, 1984: SMNS 17928), and likely *Lamplughsaura dharmaramensis* (Kutty et al., 2007: ISI R257) and *Ruehleia bedheimensis* (Galton, 2001: MB RvL 1) plot close to the *Massospondylus* growth trajectory. *Sarahsaurus aurifontanalis* (personal observation: TMM 43646-56; Marsh & Rowe, 2018: TMM 43646-2, 43646-3), the juvenile *Plateosaurus trossingensis* specimen (Nau et al., 2020: MSF 15.8B), as well as adult *Mussaurus* (Otero & Pol, 2013: MLP 68-II-27-1A) and *Qianlong* (Han et al., 2024: GZPM VN001) also plot within the 95% confidence interval of the *Massospondylus* growth trajectory. All other taxa except *Lufengosaurus huenei* (Young, 1941: IVPP V15) and *Lessemsaurus sauropoides* (Pol & Powell, 2007: PVL 4822) plot below *Massospondylus* and, overall, indicate variable scapular width within and between those taxa included here. Rather interestingly, the *Mussaurus* hatchling (Otero et al., 2019: PVL 4068) plots below *Massospondylus* individuals of similar ontogenetic stage and the adult *Mussaurus* (Otero & Pol, 2013: MLP 68-II-27-1A) is much more typical of *Massospondylus* of comparable maturity. There also appears to be noticeable taxonomic variation for the distal scapular blade width, as the majority of taxa plot below the 95% confidence interval of the *Massospondylus* growth trajectory except for *Saturnalia* (Langer, 2003; Langer et al., 2007: MCP 3844-PV, MCP 3845-PV) and *Lessemsaurus sauropoides* (Pol & Powell, 2007: PVL 4822), which plot well above (Fig. 5C). *Plateosaurus trossingensis* (Nau et al., 2020: MSF 15.8B, *Yunnanosaurus huangi* (Young, 1942: IVPP V20), and likely *Lamplughsaura dharmaramensis* (Kutty et al., 2007: ISI R257) plot within the 95% confidence interval of the *Massospondylus* growth trajectory. The *Mussaurus* hatchling and adult (Otero & Pol, 2013: MLP 68-II-27-1A; Otero et al., 2019: PVL 4068) plot uniformly below the 95% confidence interval of the *Massospondylus* growth trajectory, while the embryo and adult *Qianlong* (Han et al., 2024: GZPM VN001, GZPM VN004-2) plot within or on the bottom fringe. *Buriolestes schultzi* (Müller et al., 2018: CAPPA/UFSM 0035) and *Eoraptor lunensis* (Sereno et al., 2013: PVSJ 512) consistently plot below 95% confidence interval of the *Massospondylus* growth trajectory for all scapular growth trajectories.

The tibia of most early-diverging sauropods included in this analysis plot outside the 95% confidence interval of the *Massospondylus* growth trajectory, except for the *Mussaurus* hatchlings (Otero et al., 2019: PVL 4068; Otero & Pol, 2021: MACN-PV 4111) and the *Qianlong* embryo (Han et al., 2024: GZPM VN004-2), which plot well within this 95% confidence interval and remain close to or within it for more mature individuals (Fig. 4B). *Plateosaurus trossingensis* (Nau et al., 2020: MSF 15.8B) as well as *Adeopapposaurus mognai* (Martínez, 2009: PVSJ610+PVSJ569) also plot within the confidence interval but the rest of the taxa plot above or beyond the confidence interval. It is likely that many taxa like *Sellosaurus gracilis* (Galton, 1984: SMNS 17928)*, Riojasaurus incertus* (Rauhut et al., 2011: PVL 3808)*,* and *Lamplughsaura dharmaramensis* (Kutty et al., 2007: ISI R257) also would plot within or very close to the *Massospondylus* growth trajectory, if larger individuals of the latter are discovered. Interestingly, *Anchisaurus polyzelus* (Galton, 1976: YPM 1883), *Lufengosaurus huenei* (Young, 1941: IVPP V15), *Plateosaurus gracilis* (Huene, 1907/1908: SMNS 53537), and *Lessemsaurus sauropoides* (Pol & Powell, 2007: PVL 4822) all plot underneath the 95% confidence interval of the *Massospondylus* growth trajectory, suggesting a shorter tibia relative to the femur length.

## Discussion

### *Massospondylus* embryos and eggshells

The previously described *Massospondylus* embryos of BP/1/5347 have most recently been estimated at about 60% through incubation based on the degree of cranial ossification (Reisz et al., 2005, 2010; Chapelle et al., 2020a). Even without complete cranial material, our growth series finds the new embryos of BP/1/5346 to be a slightly more advanced ontogenetic stage than those of BP/1/5347 (Fig. 3). As such, the new embryonic materials of BP/1/5346 and their respective eggshells present a unique opportunity to examine the phenomenon of ontogenetic change in early dinosaur eggshells (Stein et al., 2019; Halgrain et al., 2022; Han et al., 2024).

These eggshells from BP/1/5346 show the same largely undifferentiated crystalline calcite morphology as those of the previously published eggshell material BP/1/6229 and BP/1/5347 from the same site and appear to exhibit similar diagenetic modification (Reisz et al., 2012; Stein et al., 2019). The preservation of skeletal material of the two clutches BP/1/5346 and BP/1/5347 are also identical, indicating the consistent depositional environment of this locality, and the similar, likely identical preservational conditions. In BP/1/5347, histological examination has revealed that the mammillary cones were merged with, or are entirely obscured by the overlying isotropic layer (Stein et al., 2019: Fig. S3). As such, even though we are unable to histologically sample BP/1/6229 or BP/1/5346, the overall similarities between these and other eggshell materials that have been collected from this site allow us to compare their thicknesses. The eggshell from eggs without embryos, BP/1/6229 (Supplementary File 2: Fig. S5), have an eggshell thickness ranging from 140-190 µm, the embryo BP/1/5347A has a thickness ranging from 80-100 µm (Stein et al., 2019), while the eggshell of the slightly larger and more developed embryos of BP/1/5346 measure roughly 38–56 µm in thickness (Fig. 1E). Thus, together with previous embryonic and eggshell material of *Massospondylus* from the same site, we observe an incremental range of eggshell thicknesses likely reflecting levels of eggshell resorption that appear to correlate with the development of their respective embryos, just as in birds (Halgrain et al., 2022). Even though we do not have fossils representing a complete embryonic ontogeny, the current ontogenetic stages are sufficient to raise the possibility that the eggs with more developmentally advanced embryos exhibit greater degrees of eggshell resorption and vice versa. This raises the critical possibility that eggshell thickness and hardness can only be evaluated with confidence in eggs lacking embryos, which adds a new level of complexity to the dinosaur eggshell debate and should be considered in future research.

### Inferring postures of *Massospondylus*

The primitive condition of adult dinosaurs appears to be bipedalism (Sereno, 1997), but reversion to secondary quadrupedality from the primitive bipedal condition appears to have occurred at least four times within Dinosauria, and includes ceratopsians, ornithopods, thyreophorans, and most relevant to our results, sauropods (Sereno, 1997; Barrett & Maidment, 2017; Maidment et al., 2014). Among extinct dinosaurian taxa that showcase a spectrum of locomotory abilities, a recent example is *Scutellosaurus lawleri* (Anderson et al., 2023). Several other dinosaurs have also apparently undergone some form of ontogenetic postural change that may have been related to paedomorphosis (e.g., Norman, 1980; Heinrich et al., 1993; Dilkes, 2001; Zhao et al., 2013). *Massospondylus* is one such dinosaur suggested to have undergone a ontogenetic postural change from a quadruped to a biped (Reisz et al., 2005, 2010, 2012; Bonnan & Senter, 2007; Yates et al., 2010).

Recently, several studies have sought to test if this dinosaur was quadrupedal at the earliest ontogenetic stages (Reisz et al., 2005, 2010, 2012; Neenan et al., 2019; Chapelle et al., 2020b, 2022). Some studies aimed at inferring the postures of *Massospondylus* have used specific anatomical proxies to argue against a postural shift (Neenan et al., 2019; Chapelle et al., 2020b, 2022). The first approach involves mapping of the inner ear labyrinth, with specific focus on the height of the vertical canal, as a method for inferring postures in dinosaurs (Georgi et al., 2013; Neenan et al., 2019). However, the reliability of this approach has also been seriously questioned (Taylor et al., 2009; Marugán-Lobón et al., 2013; Benson et al., 2017; Neenan et al., 2019). The ontogenetic changes of the inner ear of *Massospondylus* were reconstructed (Neenan et al., 2019), and although there may be slight ontogenetic variation in the vertical canal, it does not firmly support or reject postural change (Georgi et al., 2013; Neenan et al., 2019). Nevertheless, Neenan et al. (2019) argue that the consistent  “alert” head posture of *Massospondylus* throughout ontogeny does not suggest a postural change (Neenan et al., 2019). However, the nature of this postural shift debate is not that of a postural change of the head, but that of the entire body, and while the head is not completely unrelated to the posture of an animal, it cannot be solely relied upon as an indicator of overall posture.

The second approach aimed to infer postures is based on humeral and femoral circumference using a sample of largely extant mammals (Chapelle et al., 2020b). This method is well suited for inferring habitual postures in most extant animals whose postures are uncontroversial; however, we stress that this analysis does not confidently reflect the posture of *Massospondylus* at early ontogenetic stages. Chapelle et al. (2020b) rely only on two relevant individuals to reject a hypothesized postural shift in *Massospondylus*. The first is the embryo BP/1/5347A, which they recovered in their analysis as having an equivocal posture, and the second is the hatchling SAM-PK-K413, which they recovered as a biped. We caution against this finding that hatchling individuals of *Massospondylus* are likely bipedal, because it relies entirely on the humeral circumference of SAM-PK-K413, which is missing much of the midshaft (Fig. 2).

A final approach uses cyclical growth marks (CGM) and lines of arrested growth (LAG) from the midshafts of the limb bones of *Massospondylus.* Chapelle et al., (2022) argue against a postural shift in *Massospondylus* on the basis of non-differential growth rates of the forelimbs relative to the hindlimbs throughout ontogeny. Most importantly, however, this analysis lacks sufficient representation of individuals from the ontogenetic stages when this hypothesized postural shift is likely to have occurred. It relies mainly on adult and subadult individuals, and synchrotron radiation-based micro-computed tomography (SRμCT) data of the embryo BP/1/5347A. In particular, we caution the reliability of this SRμCT embryonic data because it does not permit recognition of CGMs and LAGs for a confident estimation of growth rate (Chapelle et al., 2022). Contrary to these findings by Chapelle et al. (2022), our growth trajectory for *Massospondylus* (Fig. 3) clearly shows differential growth rate of the forelimbs relative to the hindlimbs (excluding the metapodials), wherein the hindlimbs lengthen at a higher rate than the forelimbs, which is consistent with a postural shift.

**Fig. 6:**
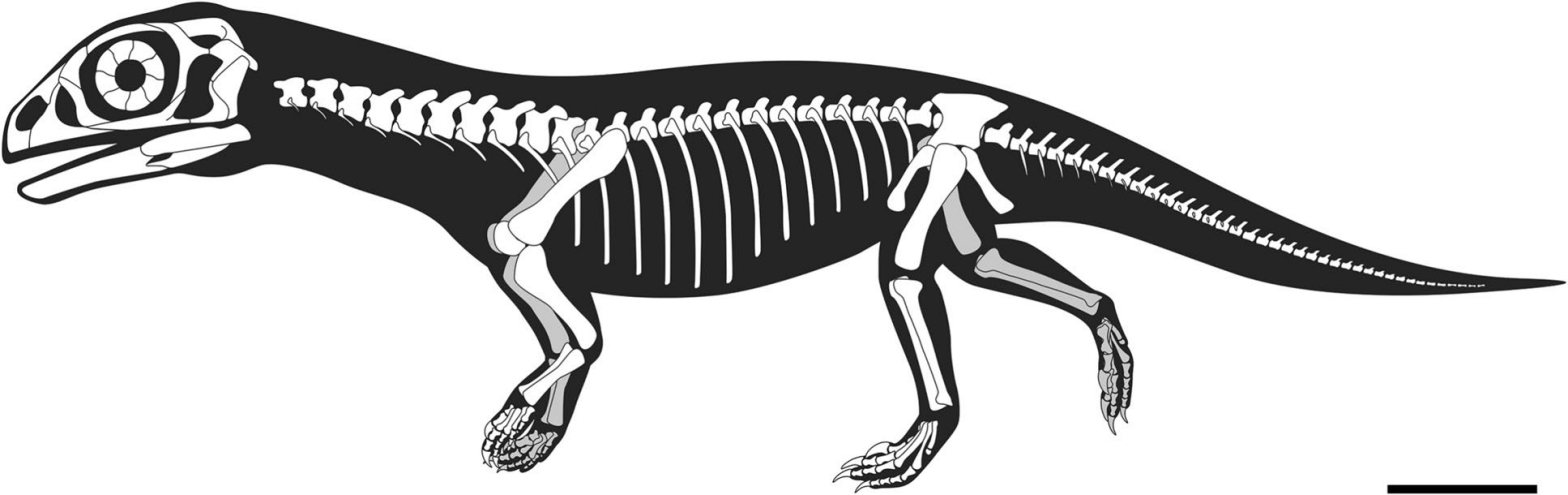
*Massospondylus* embryo quadrupedal walking reconstruction. Modified by Heidi Richter from the original Reisz et al. (2005). Scale 1 cm.

Conversely when inferring the possible postures of extinct animals, it is critical to consider the anatomy of the animal as a whole, and applying this rationale to *Massospondylus* appears to support a postural shift. Consistent with previously described embryos of BP/1/5347, the proportionately large head and elongated cervical vertebrae reflect a long neck with a heavy skull as in the other early-diverging sauropodomorphs *Mussaurus* (Otero & Pol, 2021) and *Qianlong* (Han et al., 2024) (Fig. 6). The newly discovered embryonic caudal series of BP/1/5346 (Fig. 1B) indicates that the caudal vertebrae of *Massospondylus* are small and slender, with small transverse processes and hemal spines consistent with the presence of a gracile tail (Reisz et al., 2005, 2010). As in *Mussaurus, Qianlong* and living quadrupedal mammals and reptiles, these proportions indicate that the centre of mass the embryos (BP/1/5346 & BP/1/5347) and the hatchling (SAM-PK-K413) is well forward of the pelvic gridle and necessitates a quadrupedal posture (Reisz et al., 2005, 2010; Allen et al., 2013; Bates et al., 2016; Otero & Pol, 2021). The reconstructed anatomy of the embryonic skeleton shows this set of skeletal proportions (Fig. 6), and it is thereby unlikely that newly hatched hatchlings could have maintained an effective bipedal posture. These ontogenetically influenced body proportions are most appropriately interpreted as indicators of transitional stages along a continuum of postural change, as has similarly been proposed recently for other early sauropodomorphs (Otero et al., 2019; Chapelle et al., 2020b; Otero & Pol, 2021; Creda et al., 2022; Han et al., 2024). This postural continuum would shift from obligate quadruped for newly hatched individuals*,* to facultative bipeds, to bipeds who are facultative quadrupeds, and eventually obligate bipeds for mature individuals, once a reduced forelimb length dictated such a locomotory system and posture.

Both embryos of BP/1/5346 and the hatching SAM-PK-K413 lack ossified carpals, tarsals, and have poorly ossified articulating surfaces of the limb bones to facilitate rapid growth and elongation of these bones post-hatching (Starck, 1996, 1998; Kundrát et al., 2008). As such, they are also much more flexible than those of subadults and adults, which allows for a greater range of motion than in the latter and can make interpreting the critical range of motion of the forelimb and manus tenuous (Otero et al., 2017). Just like *Mussaurus* and *Lufengosaurus huenei*, the *Massospondylus* hatchling SAM-PK-K413 and just-hatched individuals of BP/1/5346 and BP/1/5347 were likely altricial, remaining close to or within the nest, and not necessarily relying on effective quadrupedal locomotion (Reisz et al., 2005, 2010, 2013, 2024; Bonnan & Senter, 2007; Brusatte et al., 2010; Otero et al., 2019; Otero & Pol, 2021). Trackways at the Rooidraai nesting site attributed to *Massospondylus* hatchlings also support a quadrupedal posture for this ontogenetic stage (Reisz et al., 2012).

Given the lack of articulating surfaces in such immature individuals, posture must be inferred using several alternative anatomies. The in situ articulation of the left forearm of SAM-PK-K413 remains in the pronated position with the radius craniomedially oriented with the ulna, which may indicate a possible quadrupedal stance (Bonnan, 2003; Otero & Pol, 2021). This orientation contrasts with that of mature bipedal *Massospondylus* individuals (e.g., BP/1/4376) that show the opposite condition of a craniolaterally oriented radius (Bonnan & Senter, 2007). Other anatomies suggested to indicate pronation of the manus, like the anterolateral process of the ulna and distal facet of the radius, are also not observable (Yates & Kitching, 2003; Yates et al., 2010). Furthermore, the femur is clearly straighter and less sinusoidal than that of confidently bipedal sauropodomorphs (Yates et al., 2010), which may indicate a more quadrupedal posture, as it is intermediate to that of the straight columnar sauropod condition and that of clearly bipedal earliest diverging sauropodomorphs like *Eoraptor lunensis* (Sereno et al., 2013). The scapular blade may also be important in considerations of posture for these early dinosaurs, because the height of the scapular blade may influence the position of the glenoid fossa on the side of the chest and the width of the distal scapular blade is related to attachment of relevant forelimb musculature. It is notable that the *Mussaurus* and *Qianlong* hatchlings both have similarly long and slender scapulas that plot just below the confidence interval for the growth trajectory of *Massospondylus,* while the adults plot well within the pattern shown by the latter (Fig. 5). A similarly long and slender scapula is also seen in titanosaur hatchlings (GSI/GC/2904), which are unambiguously considered quadrupedal sauropods (Wilson et al., 2010). Conversely, it is interesting to note that a more distally expanded scapular blade is typical of the clearly bipedal earliest diverging sauropodomorphs like *Eoraptor lunensis* (Sereno et al., 2013) and mature *Massospondylus* specimens (e.g., BP/1/4934: Fig. S2). These morphologies of the scapular blade appear to correlate with distinct postures among sauropodomorphs and warrant future investigation.

Moreover, the reliability of allometric limb length methods for inferring posture within Sauropodomorpha have been questioned in light of the noticeable variation between these early-diverging taxa (McPhee et al., 2018; Rauhut et al., 2011; Chapelle et al., 2020b), which appears consistent with the general trend of forelimb lengthening towards Sauropoda (Barrett & Upchurch, 2005; Rauhut et al., 2011). As such, this method is best used in conjunction with other osteological indicators (Galton, 1970; Dilkes, 2001; Maidment & Barrett, 2014). It is worth noting that among more basal sauropodomorphs, particularly below the clade of *Melanorosaurus* + Sauropoda, more bipedal mature sauropodomorphs are suggested to have a higher discrepancy between fore and hind limb lengths and have a humerus to femur ratio typically <0.7, while those with more equivocal limb lengths typical of quadrupedal eusauropods are >0.8 (Cooper, 1981; Gauthier, 1986; Yates & Kitching, 2003). It is also particularly important to consider that these proportional interpretations are most often related to subadult and adult taxa and may not have the same impact on posture in juveniles and hatchlings.

Even though the embryos BP/1/5347A and BP/1/5346 and the hatchling SAM-PK-K413 lack cartilaginous articulating surfaces, their limb lengths are still proportionately informative. The measurable humerus to femur ratio for the *Massospondylus* embryo BP/1/5347A is 0.83, the embryo BP/1/5346 is 0.79, the hatchling SAM-PK-K413 is 0.71, the youngest known immature individual BP/1/5253 with a complete femur has a ratio of 0.62, the larger individual SAM-PK-K5135 has a ratio of 0.63, and the largest known mature *Massospondylus* BP/1/4934 is approximately 0.5. Given that some *Massospondylus* specimens lack various elements, they can nonetheless be estimated with reason using our *Massospondylus* growth series (Fig. 3). As such, the complete forelimb to hindlimb ratio (combined length of humerus and ulna versus combined length of femur and tibia) for the smaller embryo BP/1/5347A is 0.71, while that of the larger embryo BP/1/5346, the hatchling SAM-PK-K413, and the largest currently known *Massospondylus* skeleton BP/1/4934 are estimated at 0.69, 0.58, and 0.44 respectively. The hatchling SAM-PK-K413 is therefore intermediate between the quadrupedal embryonic specimens and the bipedal condition of subadults and adults, indicating that the shift toward facultative bipedalism may have occurred early post-hatching. This result is not unexpected because the hatchling skeleton SAM-PK-K413 (femur length = 38 mm) is positioned between the embryonic stages BP/1/5346 (femur length = 13.86 mm) and the youngest subadult individual SAM-PK-K5135 (femur length = 350 mm) on our *Massospondylus* growth series (Fig. 3). *Mussaurus* and *Qianlong* also display considerable ontogenetic variation in such limb proportions (relative humerus and femur lengths) and both are considered to demonstrate ontogenetic posture shifts from quadruped to biped early in their ontogenies (Otero & Pol, 2021; Han et al., 2024 ). *Mussaurus* ranges from ∼0.9 in the hatchling (PVL 4068) to ∼0.66 in an adult (MLP 68-II-27-1A), while *Qianlong* ranges from ∼0.85 (VN006-1) and ∼0.83 (VN006-2) in the embryos to ∼0.49 in the adult (GZPM VN001). Interestingly, slight differences in the proportions of the zeugopodia place the condition of *Mussaurus* closer to that of *Massospondylus,* since the combined forelimb to hindlimb ratios (excluding the poorly ossified autopodia) of each are 77.5% (PVL 4068) and 71% (BP/1/5347A), respectively.

The extensive ontogenetic series and the growth trajectory of this early-diverging sauropodomorph dinosaur indicates an ontogenetic postural continuum wherein embryos and altricial hatchlings of *Massospondylus* likely relied on an obligate quadrupedal posture (Bonnan & Senter, 2007; Reisz et al., 2005, 2010, 2024), with post-hatching individuals gradually becoming facultative quadrupeds or facultative bipeds and then eventually obligate bipeds upon maturity (Alexander, 2006; Yates et al., 2010; McPhee et al., 2018; Chapelle et al., 2020b). Establishing a firm transition point between postural shifts remains difficult and may only be remedied by the discovery of early-stage juvenile and hatchling individuals. Given that early diverging sauropodomorphs  are bipedal as adults (Alexander, 2006; Yates et al., 2010; McPhee et al., 2018; Chapelle et al., 2020b), and all known hatchling and embryonic individuals have body proportions and general anatomies consistent with quadrupedality (Bonnan & Senter; 2007; Otero & Pol, 2021; Han et al., 2024), the finding that these early ontogenetic individuals of *Massospondylus* are also likely quadrupedal is fascinating and may further support the notion that quadrupedal condition of sauropods evolved through paedomorphosis (Reisz et al., 2005).

### *Massospondylus* growth trajectory and other sauropodomorph taxa

We find that despite some missing data, generalizations can be made about the ontogeny of *Massospondylus*. Several clear trends are observable; the skull undergoes extensive negative allometry during growth, as does the forelimb, whereas the neck undergoes strong positive allometry. There is also significant positive allometry in the width of the distal scapular blade. Although perhaps the most interesting ontogenetic change in *Massospondylus* is related to the iconic thumb claw. BP/1/5346 reveals that while much of the manus and pes are unossified, the first terminal digit is already well-ossified in the embryo BP/1/5346, possibly reflecting its functional importance early in ontogeny. Interestingly, the thumb claw is rather straight in the embryo BP/1/5346, becomes slightly more curved in the hatchling SAM-PK-K413, and continues to become more robust and dramatically more curved in more mature individuals (e.g., BP/1/4934 Fig. S2B). There also appears to be proportionately greater growth of the thumb claw and that of the robustness of the first digit relative to corresponding digits in the embryo BP/1/5346. In adult *Massospondylus* individuals, the robust and strongly curved first ungual likely reflects its functions related to feeding as well as defense (Galton & Upchurch, 2004; Bonnan & Senter, 2007; Barrett & Upchurch, 2007), while the function of the straighter embryonic and hatchling condition remains unclear. However, given the probable ontogenetic postural shifts of *Massospondylus*, the thumb claw likely reflects ontogenetic variation consistent with varying behaviours of feeding and defense (Galton, 1971; Galton, 1976).

The available data also indicates that there are important similarities and few differences among various early-diverging sauropodomorph taxa. Overall body proportions appear to trend along similar growth trajectories to that of *Massospondylus*, even though there may be large differences in size, and their respective phylogenetic positions (McPhee et al., 2017; Apaldetti et al., 2021; Foth et al., 2021). The similar growth trajectories of elements influencing posture in a variety of other early-diverging sauropodomorphs included here may also indicate similar ontogenetically influenced postural shifts like that of *Massospondylus*. A particularly notable trend among these early-diverging sauropodomorphs is the shortening of the humerus relative to the femur throughout ontogeny, which is consistent with a shift to bipedalism. Of course, there is taxonomic and ontogenetic variation, but the currently available data show that all included taxa appear to follow a pattern of body and skeletal proportions that plot well within or near the known growth trajectory of *Massospondylus*. Comparisons among *Mussaurus*, *Qianlong*, and *Massospondylus* are particularly interesting because they are considered phylogenetically distant from each other but are the only early-diverging sauropodomorphs represented by both hatchlings and adults. *Mussaurus* and *Qianlong* appear to differ in having longer limbed early ontogenetic individuals, which is in line with a shift toward the quadrupedal sauropod condition, indicating that they may have retained this condition longer than *Massospondylus*. In general, body proportions of *Mussaurus* and *Qianlong* plot very close to that of *Massospondylus* at various ontogenetic stages, raising the possibility that they may have had particularly similar growth trajectories. Although speculative, we predict that if new material of *Mussaurus* and *Qianlong* is included in a construction of a growth trajectory, this may be the case.

Although there are several important similarities among many early-diverging sauropodomorphs, there are few apparent differences that may indicate a differing biology from that of the well-documented *Massospondylus*. *Buriolestes schultzi* (Müller et al., 2018: CAPPA/UFSM 0035) and *Eoraptor lunensis* (Sereno et al., 2013: PVSJ 512), the most basal presumed sauropodomorph taxa, consistently plot on the fringe or beyond all other early-diverging sauropodomorphs included in this analysis, and often represent the primitive condition. Of particular note, they have longer skulls and shorter cervical vertebrae relative to the length of the femur when compared to those of comparatively more advanced non sauropod sauropodomorphs like *Massospondylus*. Neck-cervical elongation is typical of many sauropodomorphs, but most Early Jurassic taxa plot slightly below *Massospondylus*, indicating that *Massospondylus* had longer than average cervical vertebrae relative to the femur when compared to many other well-known Early Jurassic sauropodomorphs.

Differences among taxa based on their geographies and temporal separation also reveal few apparent differences among early-diverging sauropodomorphs. Of the taxa included in this study, there may be a trend where Chinese and South African taxa have generally longer skulls, perhaps indicating a potential difference in feeding preference or behaviour. South American taxa also appear to have shorter cervical vertebrae than South African taxa, followed by American and European taxa. Interestingly, apart from *Coloradisaurus brevis* (Apaldetti et al., 2012: PVL 5904), Late Triassic taxa plot below that of *Massospondylus*, suggesting that the primitive condition may be relatively shorter dorsal vertebrae relative to the length of the femur. Otherwise, there does not appear to be any other differing trends of significance based on geography, as all taxa included in this analysis plot within proximity from one another for all other measured elements.

Although early-diverging sauropodomorph dinosaurs that extend from the Late Triassic and into the Early Jurassic do not form a clade, the ontogenetic series of *Massospondylus* and comparisons between various early-diverging sauropodomorph taxa indicate that there are strong similarities among them in terms of body proportions, posture, and likely their lifestyles. This is a surprising outcome and warrants future study, considering these early-diverging sauropodomorphs were able to achieve a global distribution while maintaining similar body plans until the emergence of the first Late Jurassic sauropods.

## Supplementary Information

Additional file 1: All sauropodomorph growth series measurements

Additional file 2: Illustrations of Massospondylus carinatus BP/1/4934 and eggshell BP/1/6229

## Data Availability

No datasets were generated or analysed during the current study.
