# Supplementary material for: Massospondylus embryos and hatchling provide new insights into early sauropodomorph ontogeny
Source: Swiss J Palaeontol. 2025 Aug 4;144(1):44. doi: 10.1186/s13358-025-00382-5 (PMC12321941; doi:10.1186/s13358-025-00382-5)
Supplement: Supplementary file 1 — Additional file 1: All sauropodomorph growth series measurements [file 13358_2025_382_MOESM1_ESM.pdf]

Supplementary Data 1

Massospondylus embryos and hatchling provide new insights into early sauropodomorph ontogeny

Ethan D. Mooney,[1,2,*] Tea Maho,[1,2] Dylan C.T. Rowe,[1,2] Diane Scott,[1] Robert R. Reisz[2,1*]

[1]Department of Biology, University of Toronto Mississauga, 3359 Mississauga Rd., Mississauga L5l1C6, Ontario, Canada
[2]Dinosaur Evolution Research Center, International Center of Future Science, Jilin University, 2699 Qianjin Str., Changchun, Jilin Province 130012, China

Measurements were taken whenever possible directly from those listed in their respective publication. If such measurements were not listed, measurements were taken directly from figures within their respective publications assuming they are shown to proper scale and may vary slightly. Of particular note, measures of the *Qianlong shouhu* embryo GZPM VN004-2 (Han et al., 2024) are properly adjusted considering the scale of supplementary figure 6b is incorrect and reads 5mm instead of 1cm.

Table 1. *Massospondylus* ontogeny measurements expanded from Reisz et al. (2005).

Specimen	Femur Length (mm)	Skull Length (mm)	Mid-Cervical Length (mm)	Mid-Dorsal Length (mm)	Humerus Length (mm)	Ulna Length (mm)	Tibia Length (mm)	Scapula Length (mm)	Minimum Scapula Shaft Width (mm)	Maximum Scapula Blade Width (mm)
BP/1/5347A	11.5	19.5	2.3	2.4	9.5	6.5	11	12	1.6	2
BP/I/5346	13.86			2.93		7.7		16	2	2.65
SAM-PK-K413	38		7.1	6.5	27.1	16.1		38	4	8
SAM-PK-K388	231	112	68	35	136	95	211			
SAM-PK-K391	248		71	38	155	112	220			
BP/1/4998	340	150		53	175	128	265			
SAM-PK-K5135	350		120	65	220	150	300			
BP/1/5241	360	170	115	60	230		320	291	45	103
BP/1/4934	550	200	150	75	275	170		411.3	56.2	136.3

Table 2. Early diverging sauropodomorph measurements.

Source	Taxon	Specimen Number	Femur Length (mm)	Skull Length (mm)	Mid-Cervical Length (mm)	Mid-Dorsal Length (mm)	Humerus Length (mm)	Ulna Length (mm)	Tibia Length (mm)	Scapula Length (mm)	Minimum Scapula Shaft Width (mm)	Scapula Blade Width (mm)
Otero & Pol, (2013)	*Mussaurus patagonicus*	MLP 68-II-27-1	700		138	100	460	257	510		84.3	145
Otero & Pol, (2013)	*Mussaurus patagonicus*	MLP 61-III-20-22	800			109			579			
Otero & Pol, (2021)	*Mussaurus patagonicus*	PVL 4068	30	33			27	18	28	27	2.5	4.5
Otero & Pol, (2021)	*Mussaurus patagonicus*	MPM-PV 1813	110						92.8			

Otero & Pol, (2021)	*Mussaurus patagonicus*	MACN-PV 4111	29.2	32	4.73	5.38	28.65	17.5	27.52			
Han et al., (2024)	*Qianlong shouhu*	GZPM VN001	748	356	115	109	370	244	590	499	84	189.5
Han et al., (2024)	*Qianlong shouhu*	GZPM VN004-2	29.6			6.2			28	29.2	1.57	5.21
Han et al, (2024)	*Qianlong shouhu*	GZPM VN006-1	29				24.8		26	26		
Han et al., (2024)	*Qianlong shouhu*	GZPM VN006-2	29			6.03	24		26			
Rauhut et al., (2011)	*Plateosaurus*	GPIT 1	440				370		440			
Rauhut et al., (2011)	*Plateosaurus*	SMNS 13200	680	342			400		490			
Huene, (1907-08)	*Plateosaurus gracilis*	SMNS 6014	788.88						516.66			
Huene, (1907-08)	*Plateosarus gracilis*	SMNS 53537	633.33		207.14		418.75		600			
Nau et al., (2020)	*Plateosaurus cf. trossingensis*	MSF 15.8B	236	150	53	36.1	121.4	79.5	211	177	22	53
Chapelle et al., (2019)	*Ngwevu intloko*	BP/1/4779	320	145	100	52	175	120	270			
Wang et al., (2017)	*Xinxiulong chengi*	LFGT-D0002	560			80						
Personal Observation	*Sarahsaurus aurifontanalis*	TMM 43646-56	413		116	64	250	164.2		272	37.5	78
Marsh & Rowe, (2018)	*Sarahsaurus aurifontanalis*	TMM 43646-2	420.43		124.1	70.16	252.35	160.7	318.6	229.14	32.1	69
Marsh & Rowe, (2018)	*Sarahsaurus aurifontanalis*	TMM 43646-3	482.76						376.37	271.66	35.66	85.33
Young, (1942)	*Yunnanosaurus huangi*	IVPP V20	450	207	105	71	231	157	360	305	50	137
Young, (1941)	*Lufengosaurus huenei*	IVPP V15	560	260	128	79	335	134	365	155	109	111
Apaldetti et al., (2012)	*Coloradisaurus brevis*	PVL 5904	422		140	68.7	324.5		494.5	281	47.5	82
Galton, (2001)	*Ruehleia bedheimensis*	MB RvL 1	788.88				377.77	294.44	688.88		81.81	
Galton, (1984)	*Sellosaurus gracilis*	SMNS 17928	495		101		323	219	435	382	49	
Galton, (1976)	*Anchisaurus polyzelus*	YPM 1883	211		48	35	150	105	145	144	18	
Müller et al., (2018)	*Buriolestes schultzi*	CAPPA/UFSM 0035	136	108.5	19.9	17.5					10	
Knoll, (2010)	*Ignavusaurus rachelis*	BM HR 20	152.7			23.74		66.2	153.63			32.7
Sereno et al., (2013)	*Eoraptor lunensis*	PVSJ 512	152	114	24	20	85	64	156	81	13	27
Langer, (2003); Langer et al., (2007)	*Saturnalia tupinquim*	MCP 3844-PV	157				97		158	111	14	92
Langer, (2003); Langer et al., (2007)	*Saturnalia tupinquim*	MCP 3845-PV	156				98		155	98	12.5	78
Martínez, (2009)	*Adeopapposaurus mognai*	PVSJ610 + PVSJ569	227						210			
Pol & Powell, (2007)	*Lessemsaurus sauropoides*	PVL 4822	782		112	80	489.26	303.28	470	624	149.71	345.14

Kutty et al., (2007)	*Lamplughsaura dharmaramensis*	ISI R257	630	98	535	360	525	542	70	180
Rauhut et al., (2011)	*Efraasia minor*	SMNS 12667	230		170		225			
Rauhut et al., (2011)	*Riojasaurus incertus*	PVL 3808	600		470		510			

References

Apaldetti, C., Pol, D., & Yates, A. (2012). The postcranial anatomy of *Coloradisaurus brevis* (Dinosauria: Sauropodomorpha) from the Late Triassic of Argentina and its phylogenetic implications. *Palaeontology, 56*, 277-301.

Chapelle, K. E. J., Barrett, P. M., Botha, J., & Choiniere, J. N. (2019). *Ngwevu intloko*: a new early sauropodomorph dinosaur from the Lower Jurassic Elliot Formation of South Africa and comments on cranial ontogeny in Massospondylus carinatus. *PeerJ, 7*, e7240.

Galton, P. M. (1976). Prosauropod dinosaurs (Reptilia: Saurischia) of North America. *Postilla, 169*, 1–98.

Galton, P. M. (1984). An early prosauropod dinosaur from the upper Triassic of Nordwürttemberg, West Germany. *Stuttgarter Beiträge zur Naturkunde, Serie B, 106*, 1–25.

Galton, P. M. (2001). Prosauropod dinosaurs from the Upper Triassic of Germany. In annonymus (Ed.), *Actas de las I Jornadas Internacionales sobre Paleontología de Dinosaurios y su entorno* (pp. 25–92). Colectivo Arqueológico y Paleontológico de Salas, Burgos, Spain.

Han, F., Yu, Y., Zhang, S., Zeng, R., Wang, X., Cai, H., Wu, T., Wen, Y., Cai, S., Li, C., Wu, R., Zhao, Q., & Xu, X. (2023). Exceptional early Jurassic fossils with leathery eggs shed light on dinosaur reproductive biology. *National Science Review, 11*, nwad258.

Huene, F. von. (1907/1908). Die Dinosaurier der europäischen Triasformation mit Berücksichtigung der aussereuropäischen Vorkommnisse. *Geologische und Palaeontologische Abhandlungen*, *Supplement-Band* 1, 1-419.

Knoll, F. (2010). A primitive sauropodomorph from the upper Elliot Formation of Lesotho. *Geological Magazine, 147*(6), 814-829.

Kutty, T. S., Chatterjee, S., Galton, P. M., & Upchurch, P. (2007). Basal sauropodomorphs (Dinosauria: Saurischia) from the Lower Jurassic of India: their anatomy and relationships. *Journal of Paleontology, 81*(6), 1218-1240.

Langer, M. C. (2003). The pelvic and hind limb anatomy of the stem-sauropodomorph *Saturnalia tupiniquim* (Late Triassic, Brazil). *PaleoBios, 23*(2), 1-30.

Langer, M. C., Franca, M. A. G., & Gabriel, S. (2007). The pectoral girdle and forelimb anatomy of the stem-sauropodomorph *Saturnalia tupiniquim* (Upper Triassic, Brazil). *Special Papers in Palaeontology, 77*, 113-137.

Martínez, R. N. (2009). *Adeopapposaurus mognai*, Gen. Et Sp. Nov. (Dinosauria: Sauropodomorpha), with comments on adaptations of basal Sauropodomorpha. *Journal of Vertebrate Paleontology, 29*(1), 142-164.

Marsh, A. D., & Rowe, T. B. (2018). Anatomy and systematics of the sauropodomorph *Sarahsaurus aurifontanalis* from the Early Jurassic Kayenta Formation. *PLoS ONE. 13*(10), e0204007.

Müller, R. T., Langer, M. C., Bronzati, M., Pacheco, C. P., Cabreira, S. F., & Dias-Da-Silva, S. (2018). Early evolution of sauropodomorphs: anatomy and phylogenetic relationships of a remarkably well-preserved dinosaur from the Upper Triassic of southern Brazil. *Zoological Journal of the Linnean Society, 184*(4), 1187-1248.

Nau, D., Lallensack, J. N., Bachmann, U., & Sander, P. M. (2020). Postcranial osteology of the first early-stage juvenile skeleton of *Plateosaurus trossingensis* from the Norian of Frick, Switzerland. *Acta Palaeontologica Polonica, 65*(4), 679-708.

Otero, A., & Pol, D. (2013). Postcranial anatomy and phylogenetic relationships of *Mussaurus patagonicus* (Dinosauria, Sauropodomorpha). *Journal of Vertebrate Paleontology, 33*(5), 1138-1168.

Otero, A., & Pol, D. (2022). Ontogenetic changes in the postcranial skeleton of *Mussaurus patagonicus* (Dinosauria, Sauropodomorpha) and their impact on the phylogenetic relationships of early sauropodomorphs. *Journal of Systematic Palaeontology, 19*(2), 1467-1516.

Pol, D., & Powell, J. E. (2007). New information on *Lessemsaurus sauropoides* (Dinosauria: Sauropodomorpha) from the Upper Triassic of Argentina. *Special Papers in Palaeontology, 77*, 223-243.

Rauhut, O. W. M., Fechner, R., Remes, K., & Reis, K. (2011). How to get big in the Mesozoic: the evolution of the sauropodomorph body plan. In N. Klein, K. Remes, C. T. Gee, P. M. Sander (Ed.), *Biology of the Sauropod Dinosaurs: Understanding the Life of Giants* (pp. 119–149). Indiana University Press.

Reisz, R. R, Scott, D., Sues, H-D., Evans, D. C., & Raath, M. A. (2005). Embryos of an early Jurassic prosauropod dinosaur and their evolutionary significance. *Science, 309*(5737), 761–764.

Sereno, P. C., Martínez, R. N., & Alcober, O. A. (2013). Braincase of *Panphagia protos* (Dinosauria, Sauropodomorpha). *Journal of Vertebrate Paleontology, 32*(sup1), 70-82.

Wang, Y-M., You, H-L., & Wang, T. (2017). A new basal sauropodiform dinosaur from the Lower Jurassic of Yunnan Province, China. *Scientific Reports, 7*, 41881.

Young, C-C. (1941). A complete osteology of *Lufengosaurus huenei* Young (gen. et sp. nov.) from Lufeng, Yunnan, China. *Palaeontologia Sinica, 121*(7), 1-53.

Young, C-C. (1942). *Yunnanosaurus huangi* Young (gen. et sp. nov.), a new prosauropod from the Red Beds at Lufeng, Yunnan. *Bulletin of the Geological Society of China, 22*, 63-104.
