# Supplementary material for: Massospondylus embryos and hatchling provide new insights into early sauropodomorph ontogeny
Source: Swiss J Palaeontol. 2025 Aug 4;144(1):44. doi: 10.1186/s13358-025-00382-5 (PMC12321941; doi:10.1186/s13358-025-00382-5)
Supplement: Supplementary file 2 — Additional file 2: Illustrations of Massospondylus carinatus BP/1/4934 and eggshell BP/1/6229 [file 13358_2025_382_MOESM2_ESM.pdf]

Supplementary Data 2

Massospondylus embryos and hatchling provide new insights into early sauropodomorph ontogeny

Ethan D. Mooney,[1,2,*] Tea Maho,[1,2] Dylan C.T. Rowe,[1,2] Diane Scott,[1] Robert R. Reisz[2,1*]

[1]Department of Biology, University of Toronto Mississauga, 3359 Mississauga Rd., Mississauga L5l1C6, Ontario, Canada
[2]Dinosaur Evolution Research Center, International Center of Future Science, Jilin University, 2699 Qianjin Str., Changchun, Jilin Province 130012, China

A

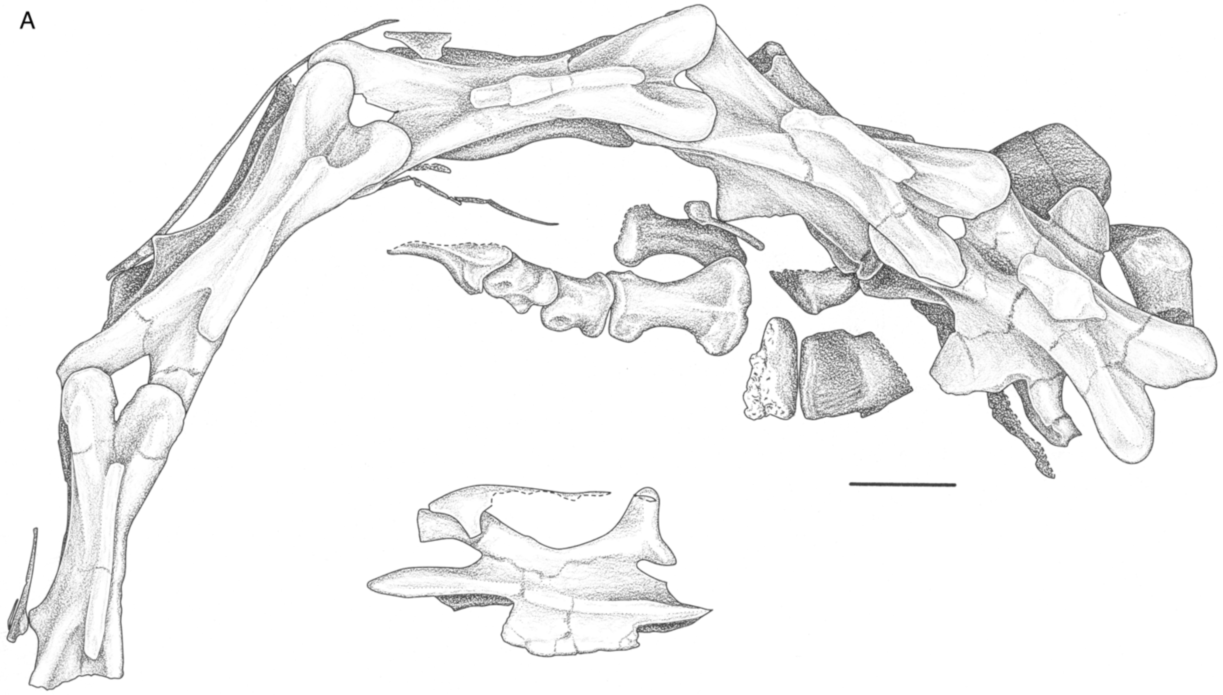

B

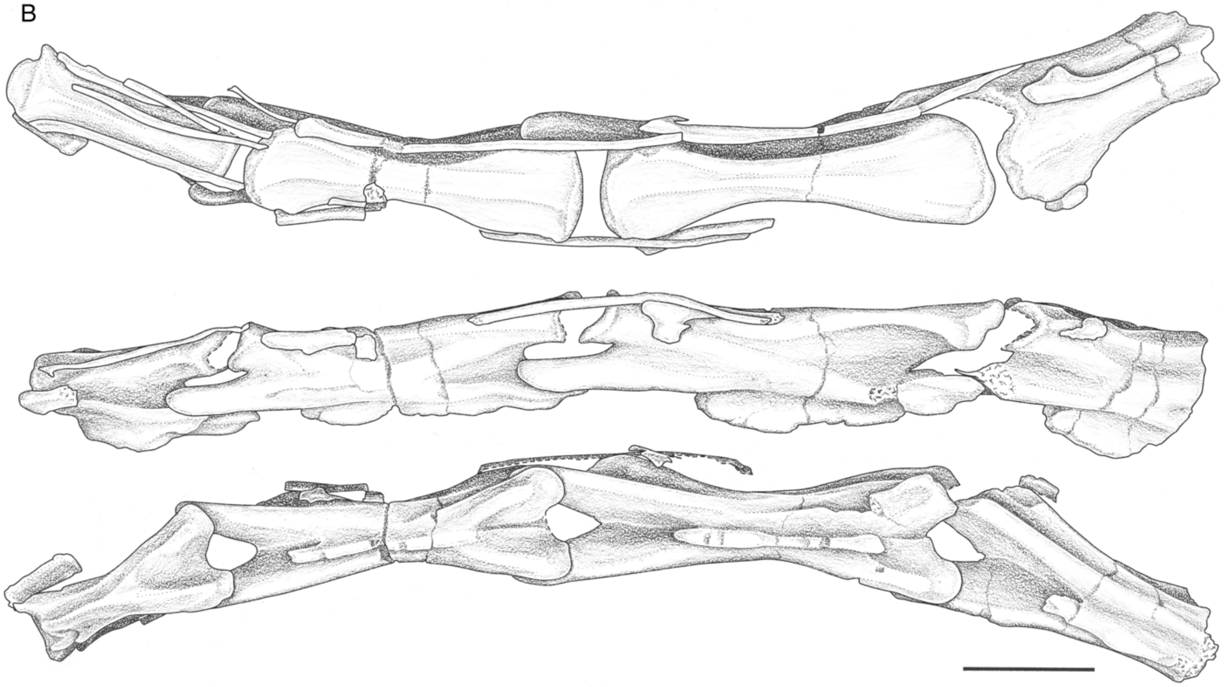

Fig. S1 *Massospondylus carinatus* (BP/1/4934) cervical region illustrations. **A** Corresponds to Barrett et al. (2019): Fig. 6; **B** Corresponds to Barrett et al. (2019): Fig. 4. Illustrations by Nikki Horseman. Scale = 5cm.

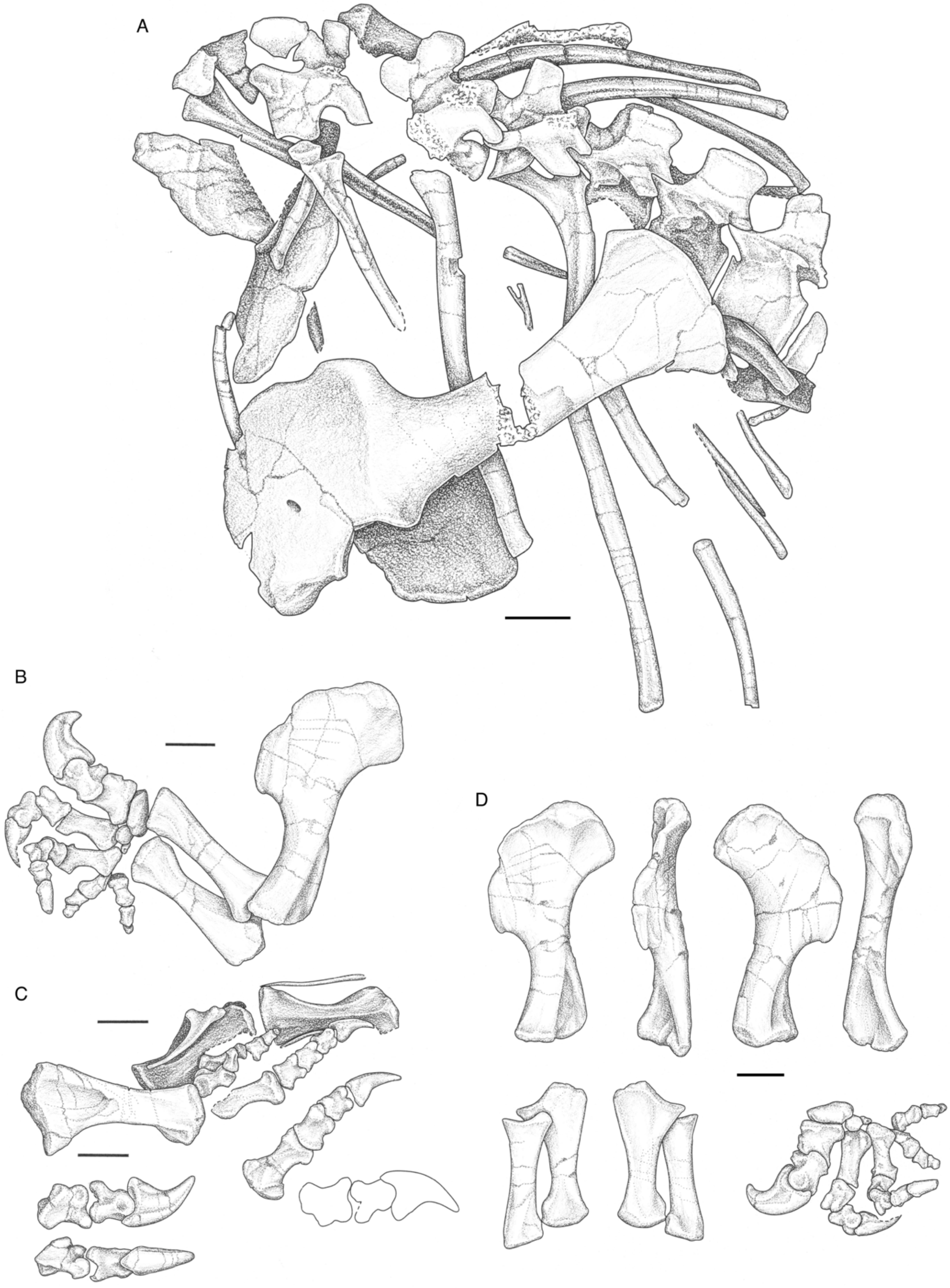

Fig. S2 *Massospondylus carinatus* (BP/1/4934) shoulder girdle and forelimb illustrations. **A** Corresponds to Barrett et al. (2019): Figs. 8, 14, 16. **B** Corresponds to Barrett et al. (2019): Figs. 14, 20. **C & D** Correspond to Barrett et al. (2019): Figs. 14, 15, 18, 19, 20. Illustrations by Nikki Horseman. Scale = 5cm.

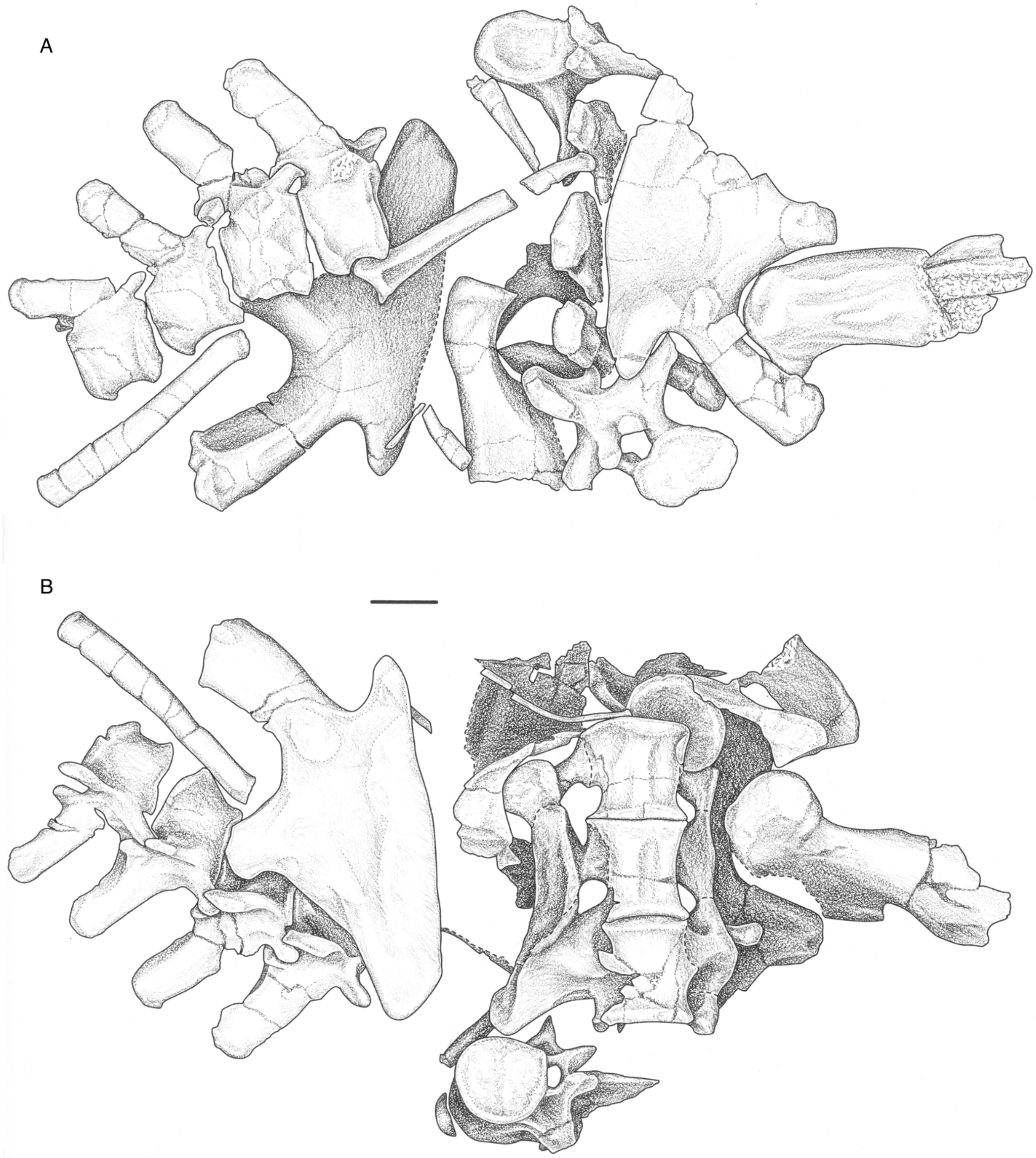

Fig. S3 *Massospondylus carinatus* (BP/1/4934) pelvic region illustrations. **A** & **B** Correspond to Barrett et al. (2019): Figs. 21, 22. Illustrations by Nikki Horseman. Scale = 5cm.

A

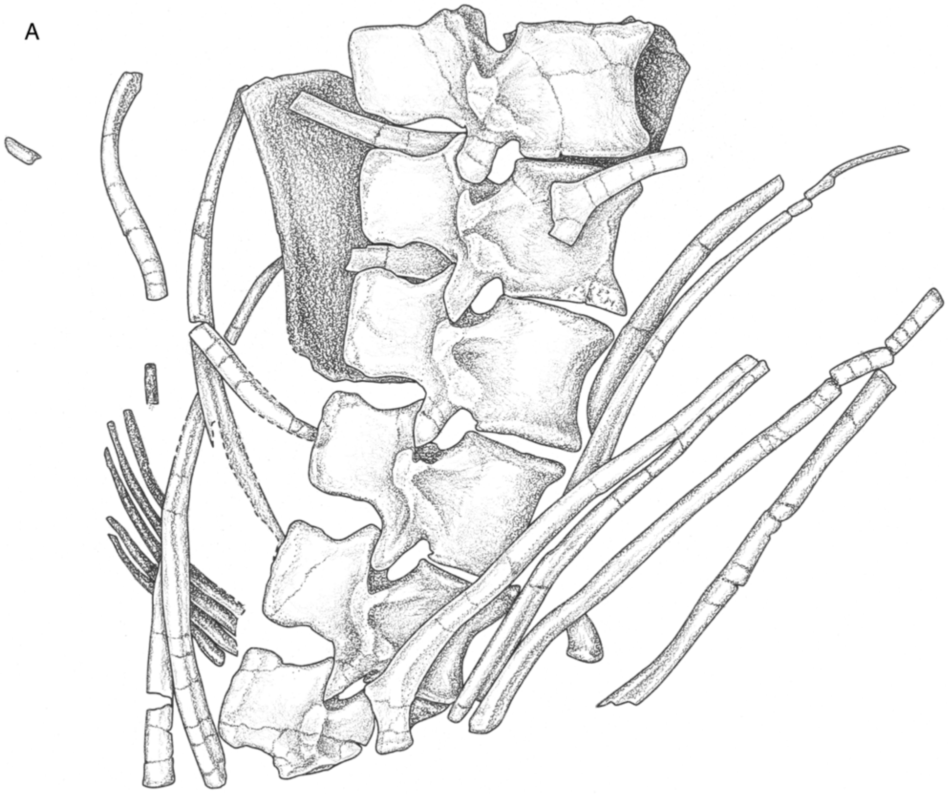

B

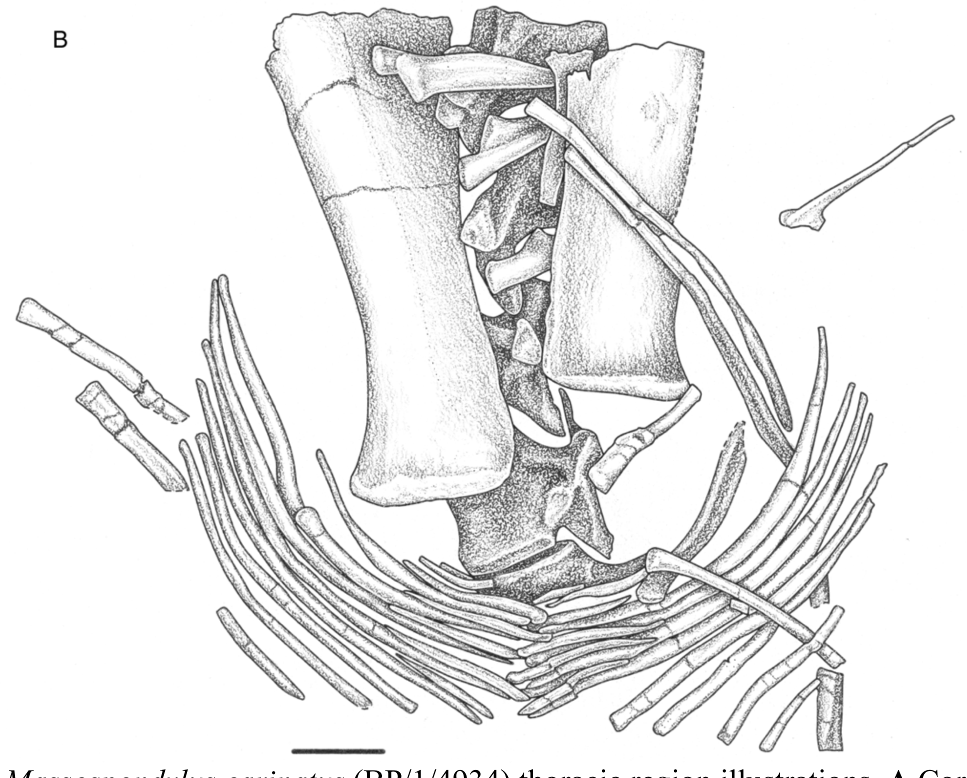

Fig. S4 *Massospondylus carinatus* (BP/1/4934) thoracic region illustrations. **A** Corresponds to Barrett et al. (2019): Figs. 9, 21. **B** Corresponds to Barrett et al. (2019): Figs. 22, 24. Illustrations by Nikki Horseman. Scale = 5cm.

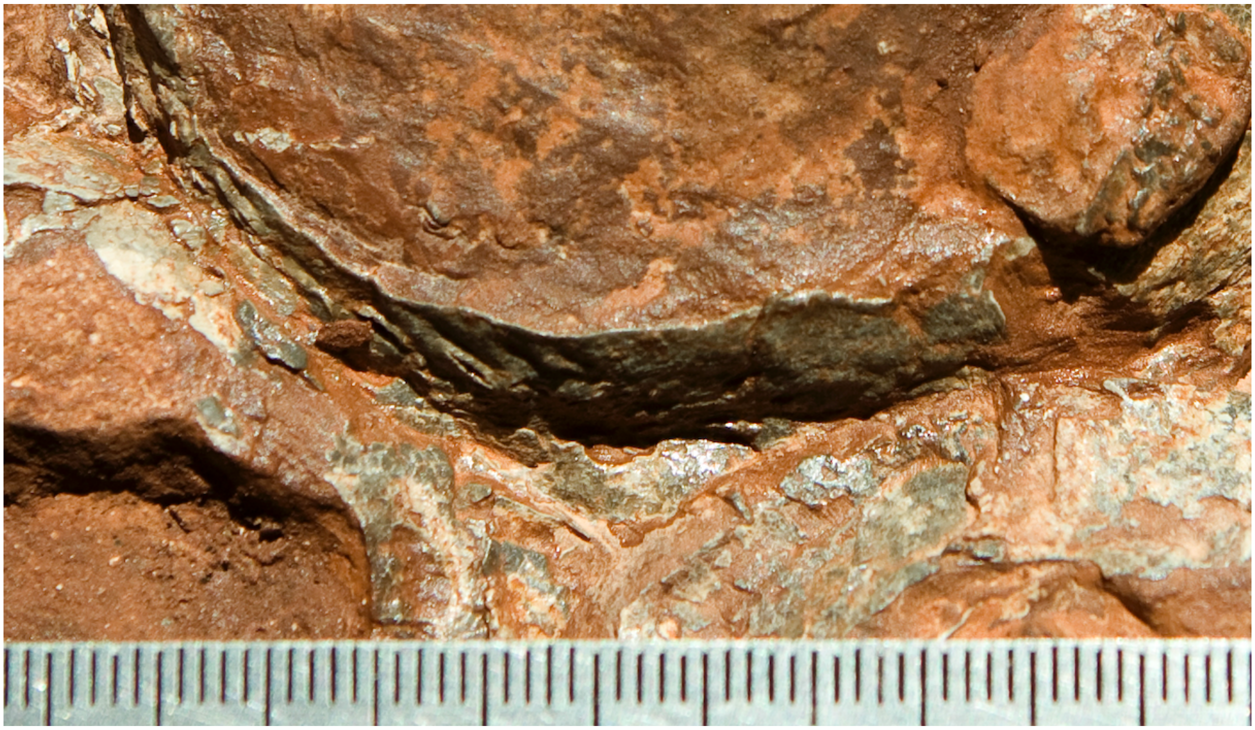

Fig. S5 *Massospondylus* eggshell BP/1/6229 lacking ossified embryonic skeletal material. Scale in mm.

References

Barret, P. M., Chapelle, K. E. J., Staunton, C. K., Botha, J., & Choiniere, J. N. (2019). Postcranial osteology of the neotype specimen of *Massospondylus carinatus* Owen, 1854 (Dinosauria: Sauropodomorpha) from the upper Elliot formation South Africa. *Palaeontologia Africana,* 53, 114-178.
